# PABPC1-induced stabilization of PGK1 mRNA reduces apoptosis and sunitinib sensitivity in renal cell carcinoma by suppressing endoplasmic reticulum stress

**DOI:** 10.1038/s41419-026-08676-3

**Published:** 2026-04-03

**Authors:** Xinran Chen, Senming Cao, Tongyu Jia, Changwei Shi, Zhenze Yang, Qiyang Liang, Xu Zhang, Liangyou Gu, Xin Ma, Qingbo Huang, Xiubin Li

**Affiliations:** 1 Medical School of Chinese PLA, Beijing, China; 2 School of Medicine, Nankai University, Tianjin, China; 3 Senior Department of Urology, Chinese PLA General Hospital, Beijing, China

**Keywords:** Cancer therapeutic resistance, Renal cell carcinoma

## Abstract

Sunitinib resistance poses a significant challenge in the management of advanced and metastatic clear cell renal cell carcinoma (ccRCC). Although RNA-binding proteins (RBPs) have recently emerged as important regulators of tumorigenesis, their roles in ccRCC progression and sunitinib resistance remain poorly understood. Through comprehensive bioinformatics analysis of clinical datasets, we identified PABPC1 as an RBP significantly upregulated in ccRCC. Functionally, PABPC1 promoted the proliferation, migration, invasion, and sunitinib resistance of ccRCC cells. Mechanistically, PABPC1 bound to and stabilized PGK1 mRNA, thereby upregulating PGK1 expression. This upregulation reduced endoplasmic reticulum (ER) stress, inhibited apoptosis, and consequently conferred sunitinib resistance in ccRCC cells. Importantly, treatment with Eeyarestatin I, a small-molecule ER stress agonist, restored sunitinib sensitivity in tumor cells. These findings reveal a novel PABPC1–PGK1 regulatory axis underlying sunitinib resistance and suggest a promising therapeutic strategy for overcoming drug resistance in ccRCC.

## Introduction

Renal cell carcinoma (RCC) is a heterogeneous malignant tumor of the kidneys, of which clear cell renal cell carcinoma (ccRCC) is the most common subtype, accounting for roughly 70% [1]. Since ccRCC is insensitive to radiation and chemotherapy, surgical resection remains the primary treatment for early-stage patients. Nevertheless, approximately 30% of patients experience tumor recurrence or metastasis after surgery [2]. Currently, combinations of tyrosine kinase inhibitors (TKIs), such as sunitinib, with or without immune checkpoint inhibitors (ICIs) represent the standard first-line treatment for metastatic RCC, offering substantial clinical benefit to patients [3, 4]. However, 10–20% of advanced or metastatic patients experience primary resistance to sunitinib, and most remaining patients eventually develop acquired resistance during therapy, limiting overall clinical efficacy [5]. Thus, clarifying the biological characteristics and regulatory mechanisms underlying sunitinib resistance is essential for improving ccRCC patient outcomes.

RNA-binding proteins (RBPs) serve as critical modulators of post-transcriptional processes, including RNA splicing, translation, transport, and localization [6, 7]. Increasing evidence has demonstrated that RBPs not only function as coordinators in normal cells but also regulate tumor development and proliferation, including in liver, breast, and colorectal cancers [8–10]. Poly(A)-binding protein cytoplasmic 1 (PABPC1), a cytoplasmic-nuclear shuttling protein expressed in most eukaryotes, is encoded by a gene located at chromosome region 8q22.2–23 [11]. As an important RBP, PABPC1 is widely involved in the initiation of translation and regulation of mRNA stability [12]. Aberrant expression and function of PABPC1 have been observed in hepatocellular carcinoma (HCC), esophageal squamous cell carcinoma (ESCC) and gliomas, contributing to aggressive tumor phenotypes [13–15]. However, the role of PABPC1 in ccRCC, particularly in the context of sunitinib resistance, remains unexplored.

Phosphoglycerate kinase 1 (PGK1) catalyzes a reversible ATP-producing step during aerobic glycolysis, converting 1,3-bisphosphoglycerate to 3-phosphoglycerate and generating one molecule of ATP to meet cellular energy demands [16]. PGK1 is frequently upregulated in various human cancers, and its activity is regulated by multiple modifications [17]. For example, under hypoxic conditions, PGK1 is aberrantly activated by hypoxia-inducible factor 1 (HIF-1), promoting tumor progression [18]. In ccRCC, PGK1 has been shown to promote tumorigenesis and sorafenib resistance by enhancing glycolytic flux [19]. However, the role of PGK1 in sunitinib resistance has not yet been investigated.

The endoplasmic reticulum (ER) is a central organelle responsible for protein synthesis, folding, post-translational modification, and calcium homeostasis, all of which are essential for maintaining cellular proteostasis [20]. Perturbations caused by extracellular or intracellular stressors, such as hypoxia, oxidative stress, and glucose deprivation, can disrupt ER homeostasis, leading to the accumulation of misfolded proteins and triggering ER stress [21, 22]. To restore equilibrium, cells activate the unfolded protein response (UPR), an adaptive signaling network [23]. However, ER stress plays a dual role in cancer: while mild or transient stress promotes cell survival and adaptation, prolonged or severe ER stress can induce apoptosis [24]. In ccRCC, sustained ER stress suppresses tumor progression and sensitizes cells to therapeutic agents, suggesting that modulating ER stress could be a strategy to overcome drug resistance.

In this study, we found that PABPC1 was significantly upregulated in ccRCC and correlated with poor patient prognosis. PABPC1 promoted ccRCC cell proliferation, migration, invasion, and sunitinib resistance. Mechanistically, PABPC1 increased PGK1 mRNA stability by interacting with the 3’ untranslated region (3’UTR). The upregulation of PGK1 inhibited ER stress activation, thereby enhancing sunitinib resistance in ccRCC. Based on these findings, treatment with Eeyarestatin I, a small-molecule ER stress inducer, effectively activated the ER stress response, leading to increased tumor cell apoptosis and restoration of sunitinib sensitivity, ultimately suppressing ccRCC progression. Taken together, our findings provide a novel therapeutic target for the treatment of sunitinib-resistant ccRCC.

## Materials and methods

### Cell cultures

Human embryonic kidney cells HEK293T, human ccRCC cell lines 786-O, OSRC-2, 769-P, ACHN were obtained from the National Platform of Experimental Cell Resources for Sci-Tech (Beijing, China) [25]. Sunitinib-resistant renal cancer cell lines were provided by the Department of Urology, Wuhan Union Hospital (786-O-R) and The First Affiliated Hospital, Sun Yat-sen University (ACHN-R). 786-O, OSRC-2, 769-P, and 786-O-R were cultured in RPMI 1640 (Procell, PM150110, China) medium with 10% fetal bovine serum (FBS) (Procell, 164210, China). ACHN and ACHN-R were maintained in MEM (Procell, PM150410, China) medium with 10% FBS. All cells were cultured in an incubator at 37 °C with 5% CO_2_ under mycoplasma-free conditions.

ER-Tracker Red was obtained from Beyotime Biotechnology, China. Sunitinib (Cat# HY-10255A), Eeyarestatin I (Cat# HY-110078) and Tauroursodeoxycholic Acid (TUDCA, Cat# HY-19696A) were purchased from MedChemExpress (MCE, USA) and solubilized in DMSO.

### Construction of sunitinib-resistant RCC cell lines

Sunitinib-resistant 786-O and ACHN cell lines were constructed through a stepwise dose-escalation protocol as described previously with minor modifications [26]. In brief, starting at 5 µM sunitinib, cells were exposed to the drug for 24 h during logarithmic growth, followed by recovery in drug‑free medium. This pulse‑treatment cycle was repeated until stable proliferation was achieved under 5 µM sunitinib. The concentration was then progressively increased to 7 µM, and the adaptation process was repeated. Successful establishment of resistance was confirmed by measuring the IC50 values of the resulting cell lines.

### Tissue samples

A total of 115 ccRCC samples and adjacent normal tissue specimens between January 2015 and December 2017 were collected to construct tissue microarrays. 8 pairs of fresh ccRCC and adjacent normal tissues, as well as 6 sunitinib-sensitive and 6 sunitinib-resistance samples were collected from patients with ccRCC, which were primarily frozen in liquid nitrogen for RNA and protein extraction. Informed consent was obtained from all patients and the Institutional Review Board of PLA General Hospital approved the implementation of this study.

### Plasmids and transfection

We designed short hairpin RNAs (shRNAs) targeting PABPC1 and PGK1, and generated knockdown constructs by cloning the corresponding double‑stranded oligonucleotides into the PLKO.1 vector. The specific sequences are listed in Supplement Table 1. The human PABPC1 and PGK1 cDNA were synthesized by BIOMED, which were cloned into pLV3-CMV-3xMyc vector to construct overexpression plasmid. Lentivirus was produced by transfection of HEK-293T cells with the constructed plasmids, and the packaging DNA, psPAX2 and pMD2-VSVG, at a 4:3:1 ratio using jetPRIME Transfection Reagent (Polyplus, France) according to the manufacturer’s instructions. The viral supernatant was collected at 48 h and 72 h, respectively. Then, we added these lentiviral particles to the target cells with polybrene (2 μg/mL, Solarbio, IPA12940, China). Puromycin (2 μg/mL, Solarbio, IP12803, China) was used to treat cells for three days to establish stable cell lines.

### Western blot (WB) analysis

We performed protein extraction using RIPA buffer (Solarbio, R0010, China) supplemented with protease and phosphatase inhibitors (Solarbio, P1260, China), as well as PMSF (Solarbio, P0100, China). The extracted protein was subjected to SDS-PAGE and transferred onto a polyvinylidene fluoride (PVDF, Millipore, USA) membrane. The membrane was blocked with 5% skim milk for 1 h at room temperature and then incubated overnight at 4 °C with primary antibodies. The next day, the membranes were washed with 1× TBST and incubated with secondary antibodies for 1 h at room temperature. Finally, the protein bands were visualized using ECL substrate kit via Tanon 5200 Imaging System. The antibodies used for western blots were as follows: PABPC1(1:5000, Proteintech, 66809-1-Ig), P-IRE1(1:1000, abclonal, AP0878), IRE1(1:2000, Proteintech, 27528-1-AP), P-PERK(1:1000, abclonal, AP0886), PERK(1:1000, Proteintech, 20582-1-AP), ATF6(1:2000, Proteintech, 24169-1-AP), GRP78(1:5000, Proteintech, 11587-1-AP), Cleaved Caspase-3(1:1000, abclonal, A27145), Cleaved PARP(1:12000, abclonal, A27147), PGK1(1:5000, Proteintech, 17811-1-AP), GAPDH(1:20000, Proteintech, 81640-5-RR).

### Immunohistochemistry (IHC)

We performed IHC for human and murine tissue samples as described earlier. The staining intensity (negative = 0, weak = 1, moderate = 2, strong = 3) and fraction of positive cells (0% – 25% = 1, 26%–50% = 2, 51%–75% = 3, ≥76% = 4) was assessed using the German semi-quantitative scoring system. The scores for intensity and the proportion of stained cells were multiplied to obtain the final score, which ranged from 0 (minimum score) to 12 (maximum score).

ER-Tracker Red was used to visualize the ER according to the manufacturer’s instructions. Next, we used CLSM 600 confocal laser scanning microscope (Sunnysoptop) to capture images and ImageJ software to process and quantify these acquired images.

### Wound healing assay

ccRCC cells were grown to confluence in 6-well plates in serum-free medium and a 200 μl pipette tip was used to generate a wound. Then, we washed 6-well plates with PBS to remove cell debris. Images were obtained at different time points (0 and 24 h) after scraping. ImageJ software was used to quantify the wound closure by measuring the remaining open wound area.

### Cell viability and apoptosis assay

2 × 10^3^ ccRCC cells were inoculated in a 96-well plate and then a Cell Counting Kit-8 (CCK-8) reagent (Abbkine, China) was added to each well to test the cell proliferation capacity at 0, 24, 48, 72, and 96 h according to the manufacturer’s instructions. The proliferation index for cells was calculated and plotted. Apoptosis assay was conducted by flow cytometry using Annexin V-PE/7-AAD staining kit (BD Biosciences, USA) following the manufacturer’s protocol, and data analysis was performed by FlowJo software.

### Transwell assay

To conduct a transwell assay, the cells were first cultured in serum-free medium for 24 h, after which they were seeded in transwell chambers with an 8-μm pore polyethylene terephthalate filter membrane (Corning, USA). To assess cell invasion, the upper chamber was coated with Matrigel (Lablead, MG2263, China) diluted at a 1:8 ratio prior to cell seeding. The cells were fixed with 4% paraformaldehyde and stained with 0.05% crystal violet after 24 h. Then, we randomly selected 5 fields that cells invaded through the membrane for cell counting.

### 5-Ethynyl-2’-deoxyuridine (EdU) assay

According to the manufacturer’s protocol, EdU staining was performed using EdU Imaging Kits (Cy3) (APExBIO, K1075, USA). Briefly, ccRCC cells were seeded on glass slides and labeled with a range of EdU concentrations (5, 10 and 20 μM) for 2 h at 37 °C. Then, these cells were fixed with 4% paraformaldehyde for 10 min and permeabilized with 0.5% Triton X-100 for 20 min at room temperature. Subsequently, these cells were exposed to staining solution for 30 min at room temperature to complete the click reaction. Finally, the cells were subjected to nuclear staining with DAPI for 15 min.

### RNA preparation and qRT‑PCR

Following the manufacturer’s instructions, total RNA extraction from either clinical samples or cultured cells was performed by using FastPure Cell/Tissue Total RNA Isolation Kit (Vazyme, RC112-01, China). Next, ND100C instrument (MIULAB, China) was used to detect concentration and purity for each RNA sample. RNA was reverse-transcribed to obtain cDNA using HiScript IV All-in-One Ultra RT SuperMix for qPCR (Vazyme, R433-01, China). We performed qRT-PCR using SupRealQ Ultra Hunter SYBR qPCR Master Mix (Vazyme, Q713-02, China). The mRNA levels were normalized to human peptidylprolyl isomerase A (PPIA). The primers were listed in Supplementary Table 2.

### RNA stability assay

Cells were treated with 5 μg/mL actinomycin D (MCE, Cat# HY-17559, USA) to block transcription in order to further measure the stability of PGK1 mRNA. Cells were collected at 0, 3, 6 and 12 h after addition of actinomycin D. Then, total RNA extraction and qRT-PCR were performed to measure the half-life of RNA.

### RNA immunoprecipitation (RIP)

According to the manufacturer’s protocol, the protein A/G magnetic beads (MCE, HY-K0202, USA) were incubated with anti-PABPC1 antibodies or IgG negative control antibody (Proteintech, 30000-0-AP, USA). Next, ccRCC cells were lysed and incubated with the corresponding antibody-coated beads. qRT-PCR was used to purify and measure the co-precipitated RNAs for further enrichment. The primer sequences were listed in Supplementary Table 2.

### Dual‑luciferase reporter assay

ccRCC cells were seeded into 24-well plates (5 × 10^4^ cells per well). The psiCHECK2 PGK1 3’UTR reporter vector was co-transfected with PABPC1 shRNA plasmids or overexpression plasmid to determine the 3’UTR activity of PGK1. After transfection for 48 h, the firefly and Renilla luciferase activities were measured with Dual-Luciferase Reporter Assay System kit (Beyotime, #RG088S, China). Renilla luciferase activity was normalized to the luminescence of firefly luciferase.

### RNA-protein docking

The PABPC1 protein sequences and 3’UTR of PGK1 were retrieved from the UniProt database (https://www.uniprot.org/) and the Encyclopedia of DNA Elements (ENCODE) database (http:// genome.ucsc.edu/), respectively. The sequences were submitted to the AlphaFold 3 server for interaction analysis [27]. The predicted interaction models were visualized using PyMOL [28] (PyMOL Molecular Graphics System, version 3.0.4, Schrodinger, New York, United States).

### Animal experiments

All animal experiments were in obedience with ARRIVE guidelines and approved by the Institutional Animal Care and Use Committee of the Chinese PLA General Hospital. Four-week-old male BALB/c nude mice were raised in SPF-grade animal laboratory and randomly divided into four groups for the xenograft tumor model. Stably downregulated PABPC1 or overexpressed PGK1 cells, as well as control OSRC-2 cells (1 × 10^6^ cells per mouse) were injected subcutaneously into the right flank of the nude mice. The length and width of xenografts were measured every three days using a Vernier caliper, and tumor volumes were calculated according to the formula length × width^2^ × 0.5. When tumor volume reached 100 mm^3^, these mice were treated with or without sunitinib (oral administration, 25 mg/Kg, once a day for 8 days) [29] or Eeyarestatin I (intraperitoneal injection, 2.5 mg/Kg, once a day for 10 days) [30]. When the mice were sacrificed, the tumors were excised and weighed.

### Bioinformatics Analysis

Data on the expression profiles and clinical prognosis of PABPC1 in ccRCC were sourced from the Cancer Genome Atlas (TCGA) database via cBioPortal (http://www.cbioportal.org/public-porta). PABPC1 pan-cancer expression levels were analyzed in the Tumor Immune Estimation Resource (TIMER) database (https://compbio.cn/timer1/). The correlation between PABPC1 and PGK1 expression was assessed in the Gene Expression Profiling Interactive Analysis (GEPIA) database (http://gepia.cancer-pku.cn/index.html). Gene set enrichment analysis (GSEA) was performed to explore the possible drug resistance mechanisms of PABPC1 in ccRCC using the RNA sequencing (RNA-seq). STRING (https:// string-db.org/) was used to create the interaction network of genes. P-values < 0.05 were regarded as statistically significant.

### Statistical analysis

All statistical analyses were performed with GraphPad Prism 9.0. Data were presented as the mean ± standard deviation (SD) from at least three independent experiments. The Student’s t-test or one-way ANOVA with Tukey’s post-hoc test was performed to determine the significance of intergroup differences. Kaplan-Meier method and log-rank test were used to analyze overall survival rates (OS) and progression-free survival rates (PFS). A P-value < 0.05 was considered statistically significant.

## Results

### Elevated PABPC1 expression is associated with poor prognosis and ccRCC progression

**Fig. 1:**
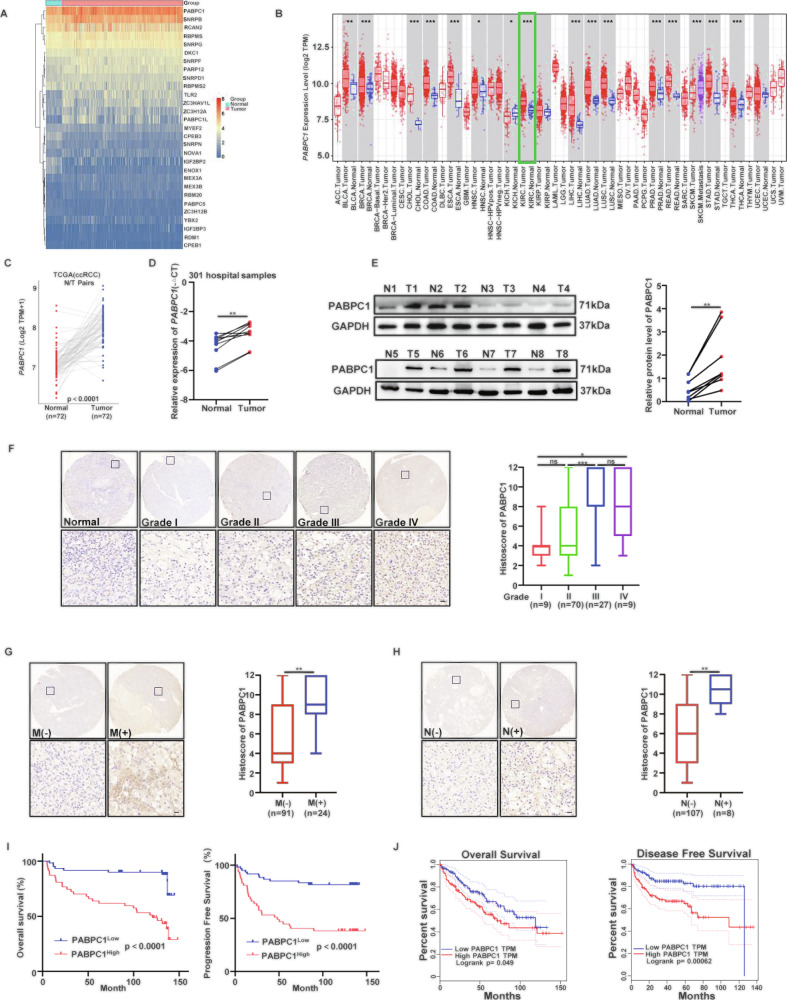
Elevated PABPC1 expression is associated with poor prognosis and ccRCC progression. **A** Different expression levels of RBPs in normal and ccRCC tissues according to the data from the TCGA database (https://cancergenome.nih.gov/). **B** The mRNA expression level of PABPC1 in various types of cancer in the TIMER database (https://compbio.cn/timer1/). **C, D** mRNA levels of PABPC1 in normal tissues and ccRCC tissues, based on the TCGA database **C** and our clinical samples **D**. **E** WB detection of PABPC1 protein expression in 8 pairs of ccRCC tissues (T) and corresponding adjacent normal tissues (N). **F** IHC staining images of PABPC1 in different tumor grades and quantitative analysis between groups with different tumor grades (I vs II, *P* = 0.474, II vs III, *P* < 0.001, III vs IV, *P* = 0.6471, I vs IV, *P* = 0.0253). Scale bar, 20 µm. **G** IHC staining images of PABPC1 in ccRCC tissues with absence or presence of preoperative distant metastases, and quantitative analysis between groups (M- vs M + , *P* = 0.002). Scale bar, 20 µm. **H** IHC staining images of PABPC1 in ccRCC tissues with absence or presence of preoperative lymph nodes metastases, and quantitative analysis between groups (N- vs *N* + , *P* = 0.007). Scale bar, 20 µm. **I, J** Kaplan-Meier curve of OS or PFS between PABPC1 high expression group and low expression group based on our tissue microarray **I** and TCGA database **J**. Data are presented as the mean ± SD of at least three independent experiments. Ns, not significant, *P < 0.05, **P < 0.01, ***P < 0.001. P values are calculated by Student’s t test or one-way ANOVA.

Analysis of RBPs using TCGA, data revealed that PABPC1 expression was elevated in ccRCC tissues compared to normal kidney tissues (Fig. 1A). Examination of PABPC1 expression across various cancers using the TIMER database showed similar trends (Fig. 1B). Additionally, bioinformatic analyses from TCGA and qRT-PCR results from our clinical samples demonstrated significantly increased PABPC1 mRNA levels in ccRCC tissues (Fig. 1C, D). Consistently, protein-level analysis indicated that PABPC1 expression was significantly higher in ccRCC tissues than in adjacent normal tissues (Fig. 1E). IHC validation using a tissue microarray comprising 115 ccRCC samples showed that PABPC1 expression positively correlated with tumor grade (Fig. 1F). Furthermore, patients with lymph node metastasis (Fig. 1G) or preoperative distant metastasis (Fig. 1H) exhibited significantly higher PABPC1 expression compared to those without metastasis. Kaplan–Meier survival analyses based on tissue microarray and TCGA data revealed that high PABPC1 negatively correlated with OS and PFS (Fig. 1I, J). Collectively, these results indicate that elevated PABPC1 expression is associated with unfavorable prognosis in ccRCC.

Given the significant upregulation of PABPC1 in ccRCC, its functional role in tumor progression was investigated. Initially, WB and qRT-PCR analyses showed higher PABPC1 expression in various ccRCC cell lines compared to HK2 cells (Figure S1A). Subsequently, stable PABPC1-knockdown and PABPC1-overexpressing ccRCC cell lines were established, and effective modulation of PABPC1 levels was confirmed (Figure S1B, C). CCK-8 assays indicated that PABPC1 knockdown significantly inhibited ccRCC cell proliferation (Figure S1D), whereas overexpression enhanced proliferation (Figure S1E). Moreover, wound healing and Transwell assays demonstrated that PABPC1 depletion markedly reduced migration and invasion of 786-O and OSRC-2 cells (Figure S1F, H, J, L), while its overexpression promoted these malignant phenotypes in ACHN and 769-P cells (Figure S1G, I, K, M). Collectively, these data confirm that PABPC1 facilitates ccRCC progression in vitro by promoting cell proliferation, migration, and invasion.

### PABPC1 reduces sunitinib sensitivity in ccRCC cells in vitro and in vivo

**Fig. 2:**
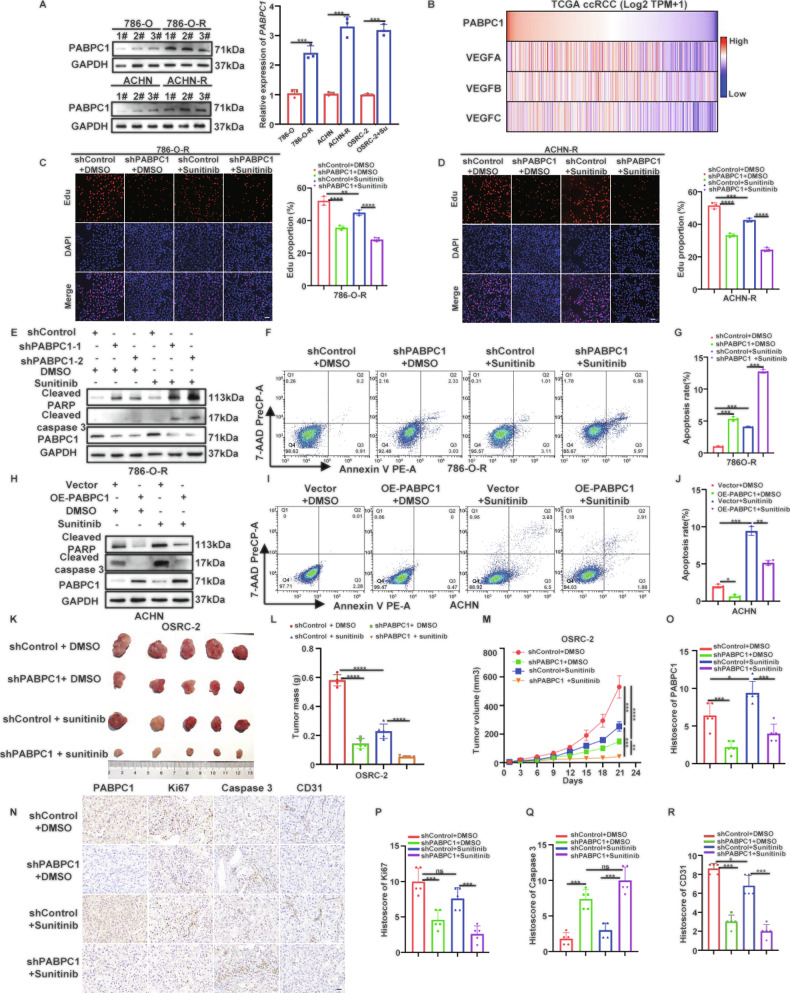
PABPC1 reduces sunitinib sensitivity in ccRCC cells in vitro and in vivo. **A** The protein and mRNA expression of PABPC1 in sunitinib‑resistant cells (786‑O-R, ACHN-R, OSRC-2 + Su) and their parental counterparts (786‑O, ACHN, OSRC‑2) were detected by WB and qRT‑PCR, respectively. **B** Heatmap of the relationship between PABPC1 and the VEGF family. **C, D** 786-O-R and ACHN-R cells were infected with lentivirus-expressing shControl or shPABPC1 for 72 h. Then, cells were treated with or without 8 µM sunitinib for 24 h. These cells were harvested for EdU assays **C**, **D** (*n* = 3 per group). Scale bar, 100 µm. **E–G** 786-O-R cells transduced with the indicated lentiviruses were treated with or without 8 µM sunitinib for 24 h. These cells were harvested for WB analysis **E** or Annexin V‑PE/7‑AAD analysis **F**, **G**. **H–J** ACHN cells were transfected with empty vector (EV) or Myc-PABPC1 for 72 h. Then, cells were treated with or without 6 µM sunitinib for 24 h. These cells were harvested for WB analysis **H** or Annexin V‑PE/7‑AAD analysis **I**, **J**. **K-M** OSRC-2 cells were infected with shControl or shPABPC1 for 72 h. After puromycin selection, cells were subcutaneously injected into nude mice for the xenograft assay. The image of tumor was shown in **K**. The tumor mass was demonstrated in L. The tumor growth curve was shown in **M**. **N–R** Representative images of IHC staining of tumor tissues from mice inoculated with shControl or shPABPC1 cells, and treated with or without sunitinib (*n* = 5 per group). Scale bar, 20 µm. Data are presented as mean ± SD. Ns, not significant, *P < 0.05, **P < 0.01, ***P < 0.001. One-way ANOVA followed by Turkey’s multiple comparisons post hoc test was applied for the statistical analysis.

Although the aforementioned results have confirmed the tumor-promoting role of PABPC1, its clinical implications in therapeutic resistance, particularly with respect to pharmacological treatments, remain unclear. Given the established clinical efficacy of sunitinib as a representative TKI for ccRCC, we examined PABPC1 expression in sunitinib-resistant cells (786-O-R, ACHN-R, and OSRC-2 + sunitinib) compared to sunitinib-sensitive cells (786-O, ACHN, and OSRC-2). Both mRNA and protein levels of PABPC1 were significantly elevated in sunitinib-resistant cells (Fig. 2A and Figure S2A). Similarly, higher PABPC1 protein expression was observed in tumor samples from sunitinib-resistant patients compared with sensitive patients (Figure S2B). Furthermore, correlation analyses based on TCGA data confirmed a positive association between PABPC1 expression and multiple angiogenic factors (Fig. 2B and Figure S2C, D), suggesting that PABPC1 might mediate sunitinib resistance in ccRCC. Moreover, we performed CCK‑8 assays to determine and compare the half-maximal inhibitory concentration (IC50) values of the resistant lines and their corresponding parental cells. The results confirmed a significant increase in drug tolerance (Figure S2E). Next, CCK-8 assays revealed that PABPC1 knockdown (Figure S2F, G) significantly decreased the IC50 values for sunitinib in 786-O-R, ACHN-R, and OSRC-2 cells (Figure S2H), whereas PABPC1 overexpression markedly increased IC50 values in ACHN and 769-P cells (Figure S2I). Additional CCK-8 and EdU assays confirmed that silencing PABPC1 reduced ccRCC cell proliferation and increased sensitivity to sunitinib compared to controls (Fig. 2C, D and Figure S2J, K). Conversely, overexpression of PABPC1 enhanced cell proliferation rates and conferred sunitinib resistance in ACHN and 769-P cells (Figure S2L-N).

Subsequently, Annexin V‑PE/7‑AAD staining and WB analyses were conducted to further evaluate apoptosis following drug treatment. Knockdown of PABPC1 significantly promoted apoptosis and increased sunitinib sensitivity in 786-O-R cells (Fig. 2E–G). Conversely, PABPC1 overexpression decreased apoptosis rates and promoted sunitinib resistance in ACHN cells (Fig. 2H–J). These findings indicate that silencing PABPC1 synergistically enhanced the anti-tumor efficacy of sunitinib. To confirm these observations in vivo, a subcutaneous xenograft tumor model was established to investigate the combined effect of PABPC1 knockdown and sunitinib treatment on ccRCC progression. Tumors derived from cells with PABPC1 silencing were significantly smaller and lighter compared to control groups, and this reduction was further enhanced by sunitinib treatment (Fig. 2K–M). Additionally, PABPC1-knockdown tumors showed decreased expression of Ki-67 and CD31, along with increased caspase-3 expression compared to controls, with these differences becoming more pronounced following sunitinib administration (Fig. 2N-R). Together, these results confirm that PABPC1 upregulation contributes to sunitinib resistance in ccRCC, both in vitro and in vivo.

### PABPC1 suppresses ER stress and reduces sunitinib sensitivity in ccRCC

**Fig. 3:**
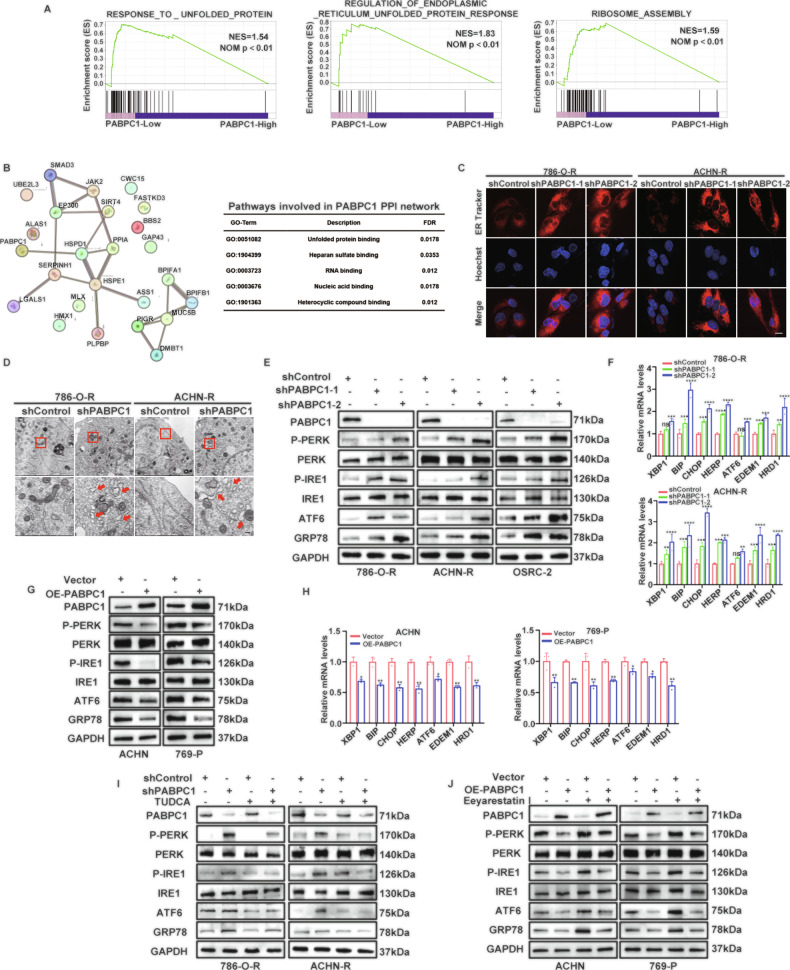
PABPC1 suppresses ER stress and reduces sunitinib sensitivity in ccRCC. **A** GSEA assays indicated the levels of PABPC1 expression were negatively correlated with regulation of UPR in ER in ccRCC according to our RNA-seq data. P-values < 0.05 were regarded as statistically significant. **B** String interaction network and pathways involved in PABPC1 PPI network. **C** Immunofluorescence technology traced ER in ccRCC cells depleting PABPC1, Red: ER-tracker, Blue: Hoechst. Scale bar, 10 µm. **D** TEM of ER stress in the control and shPABPC1 groups of 786-O-R and ACHN-R cells. Representative images with arrows indicating ER stress. Scale bar, 0.4 µm. **E, F** Protein and mRNA levels of ER stress sensors in 786-O-R, ACHN-R and OSRC-2 cell lines depleting PABPC1. **G, H** Protein and mRNA levels of ER stress sensors in ACHN and 769-P cell lines overexpressing PABPC1. **I** 786-O-R and ACHN-R cells transduced with the indicated lentiviruses were treated with or without 100 µM TUDCA for 24 h. These cells were harvested for WB analysis. **J** ACHN and 769-P cells transduced with the indicated lentiviruses were treated with or without 10 µM Eeyarestatin I for 24 h. These cells were harvested for WB analysis. Data are presented as mean ± SD. Ns, not significant, *P < 0.05, **P < 0.01, ***P < 0.001. P values are calculated by Student’s t test or One-way ANOVA.

To elucidate the precise mechanism by which PABPC1 promotes sunitinib resistance in ccRCC, we performed RNA-seq on 786-O-R and ACHN-R cells transfected with two distinct shRNAs targeting PABPC1. GSEA based on RNA-seq data indicated that knockdown of PABPC1 was strongly correlated with the response to unfolded proteins and regulation of the UPR in the ER (Fig. 3A). ER stress, characterized by the accumulation of unfolded or misfolded proteins in the ER lumen, triggers the UPR, an adaptive cellular mechanism that maintains protein homeostasis and is closely associated with sunitinib resistance in ccRCC. STRING interaction network and Gene Ontology (GO) pathway analyses further revealed that PABPC1 was associated with genes and pathways involved in protein folding and the UPR (Fig. 3B). Considering the crucial role of ER stress in modulating sunitinib sensitivity, we investigated whether PABPC1 conferred drug resistance by suppressing ER stress. ER-Tracker imaging was performed to visualize ER structural changes in cells with PABPC1 knockdown or overexpression. Notably, ER-Tracker fluorescence intensity significantly increased in PABPC1-silenced cells (Fig. 3C) and markedly decreased in cells overexpressing PABPC1 (Figure S3A). Transmission electron microscopy (TEM) further revealed morphological alterations, demonstrating significantly swollen and dilated ER cisternae with disrupted structural organization in PABPC1-silenced cells (Fig. 3D).

To validate the critical role of ER stress in ccRCC, we assessed the expression of ER stress sensors at both mRNA and protein levels. Knockdown of PABPC1 significantly increased ER stress sensors expression (Fig. 3E, F and Figure S3B), whereas PABPC1 overexpression substantially suppressed these proteins (Fig. 3G, H). Functional rescue experiments were conducted to further elucidate the role of ER stress in PABPC1-mediated sunitinib resistance. Specifically, 786-O-R and ACHN-R cells with stable PABPC1 knockdown were treated with the ER stress inhibitor TUDCA. TUDCA treatment attenuated the activation of ER stress sensors induced by PABPC1 silencing (Fig. 3I). Concurrently, the proliferation inhibition caused by PABPC1 knockdown was significantly reversed upon ER stress suppression by TUDCA (Figure S3C). In contrast, ACHN and 769-P cells with stable PABPC1 overexpression were treated with Eeyarestatin I, a small-molecule ER stress agonist that triggers ER stress by inhibiting ER-associated degradation (ERAD). Eeyarestatin I reversed the suppression of ER stress sensors caused by PABPC1 overexpression (Fig. 3J). Additionally, CCK-8 assays demonstrated that ER stress activation significantly inhibited the enhanced proliferative capacity induced by PABPC1 overexpression in ccRCC cells (Figure S3D). Collectively, these results confirm that PABPC1 suppresses ER stress, thus promoting sunitinib resistance in ccRCC.

### PGK1 is a critical factor in PABPC1‑mediated ccRCC progression

**Fig. 4:**
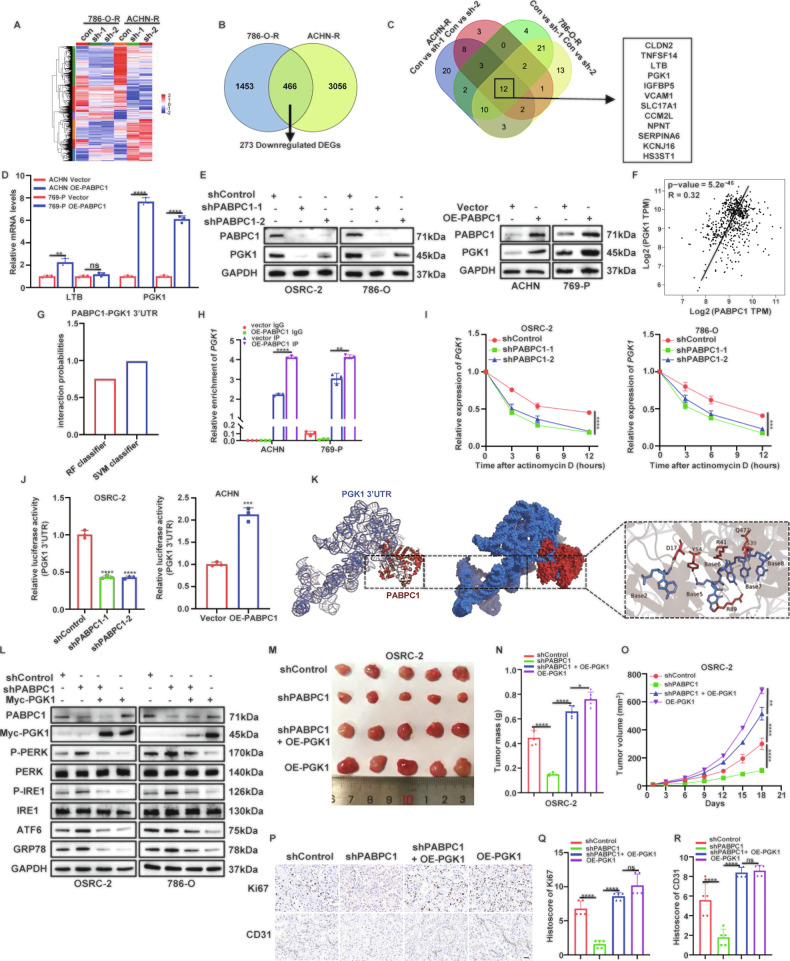
PGK1 is a critical factor in PABPC1‑mediated ccRCC progression. **A** The heatmap of cluster analysis based on the DEGs in 786-O-R and ACHN-R cells depleting PABPC1. **B** The Venn plot showing the intersection of downregulated DEGs detected by RNA-seq upon PABPC1 silencing cells. **C** The Venn plot showing 12 high‑confidence down‑regulated genes. **D** Detection of PGK1 and LTB mRNA levels by qRT-PCR upon PABPC1 overexpression in ACHN and 769-P cells (*n* = 3). **E** Detection of PGK1 protein expression by WB upon PABPC1 silencing or overexpression cells. **F** Scatter plot of the relationship between PABPC1 and the PGK1. **G** The interaction probabilities of PABPC1 with 3’UTR of PGK1 mRNA were predicted by the RPISeq website. Predictive Value > 0.5 indicated that the corresponding RNA and protein were likely to interact. **H** RIP assay, using PABPC1 antibody, demonstrating enrichment of PGK1 mRNA compared to the negative control IgG. **I** PGK1 mRNA expression levels were determined in ccRCC cells following shControl or shPABPC1 transfection after actinomycin D (5 mg/mL) treatment. **J** Luciferase activities of PGK1 3’UTR were measured after knockdown of PBAPC1 in OSRC-2 cells or overexpression of PBAPC1 in ACHN cells, respectively. **K** Virtual molecular docking of PBAPC1 and PGK1 mRNA. **L** Protein levels of ER stress sensors in indicated cell lines. **M-O** OSRC-2 cells with indicated plasmids were subcutaneously injected into nude mice for the xenograft assay. The image of tumor was shown in M. The tumor mass was demonstrated in N. The tumor growth curve was shown in O. **P-R** Representative images of IHC staining of tumor tissues from mice inoculated with indicated plasmids cells (*n* = 5 per group). Scale bar, 20 µm. Data are presented as mean ± SD. Ns, not significant, *P < 0.05, **P < 0.01, ***P < 0.001. One-way ANOVA followed by Turkey’s multiple comparisons post hoc test was applied for the statistical analysis.

To further identify downstream genes involved in PABPC1-induced ccRCC progression and sunitinib resistance, we screened transcriptome data obtained via RNA-seq (Fig. 4A), focusing on genes downregulated in 786-O-R and ACHN-R cells following PABPC1 silencing compared to control cells. The analysis revealed that 1453 and 3056 mRNAs were differentially expressed in 786-O-R and ACHN-R cells, respectively, including 273 commonly downregulated genes (Fig. 4B). To focus on the most robust candidates, we applied a dual threshold of |FC | ≥ 10 and adjusted p‑value < 0.05, yielding 12 high‑confidence down‑regulated genes (Fig. 4C). Given the established role of PABPC1 in promoting ccRCC progression and therapy resistance, we prioritized six tumor‑upregulated genes for further validation (Figure S4A). RT‑qPCR analysis confirmed that only LTB and PGK1 were significantly downregulated upon PABPC1 knockdown (Figure S4B). Strikingly, PGK1 alone exhibited reciprocal upregulation following PABPC1 overexpression (Fig. 4D). Furthermore, WB analysis verified that PABPC1 regulated PGK1 protein expression (Fig. 4E). TCGA database analysis also revealed a positive correlation between PABPC1 and PGK1 expression in ccRCC (Fig. 4F).

We next explored the mechanism by which PABPC1 regulates PGK1 expression. Previous studies have demonstrated that PABPC1 mediates mRNA stability by interacting with the 3’UTR. Therefore, we investigated whether PABPC1 enhanced PGK1 expression by stabilizing its mRNA rather than the transcriptional activity of the PGK1. qRT-PCR analysis in both PABPC1‑knockdown and PABPC1‑overexpression cell lines revealed no significant change in PGK1 pre‑mRNA levels (Figure S4C), which indicated that the observed alterations in mature PGK1 mRNA were unlikely to be driven by changes in transcription. Then, the interaction between PABPC1 and PGK1’s 3’UTR was predicted using the RNA-protein interaction prediction (RPISeq) database. Both RF classifier and SVM classifier scores exceeded 0.5, suggesting a high likelihood of interaction between PABPC1 and the PGK1 mRNA 3’UTR (Fig. 4G). Additionally, RIP assays confirmed the binding of PABPC1 to PGK1 mRNA (Fig. 4H). When ccRCC cells were treated with the RNA synthesis inhibitor actinomycin D, PGK1 mRNA half-life was significantly shorter in PABPC1-depleted cells compared to controls (Fig. 4I). Conversely, PABPC1 overexpression increased PGK1 mRNA stability by extending its half-life (Figure S4D). Furthermore, luciferase reporter assays showed that PABPC1 knockdown significantly decreased, whereas PABPC1 overexpression increased, the luciferase activity of the PGK1 3’UTR reporter (Fig. 4J). Using AlphaFold 3, we predicted potential interaction domains between PABPC1 amino acids and the PGK1 3’UTR. Results indicated that PABPC1 stabilized PGK1 mRNA by interacting with its 3’UTR (Fig. 4K).

To determine whether PGK1 mediated the biological effects of PABPC1 on ccRCC progression, PGK1 was overexpressed in PABPC1-silenced cells. Overexpression of PGK1 significantly rescued the suppressed proliferation, migration, and invasion abilities of ccRCC cells caused by PABPC1 knockdown (Figure S4E-J). Moreover, PGK1 overexpression inhibited the activation of ER stress induced by PABPC1 silencing (Fig. 4L). Subsequently, we established xenograft mouse models to evaluate the effect of PGK1 overexpression on tumor growth in PABPC1-silenced ccRCC cells. Tumor size and weight were markedly reduced in the PABPC1-silenced group, an effect reversed by PGK1 overexpression (Fig. 4M-O). IHC analysis further revealed decreased Ki-67 and CD31 expression in tumors derived from PABPC1-silenced cells, which was restored by PGK1 overexpression (Fig. 4P-R). Collectively, these findings indicate that PABPC1 protects PGK1 mRNA from degradation, and PGK1 functions as a critical mediator of PABPC1-driven ccRCC progression.

### PGK1 contributes to PABPC1-induced sunitinib resistance by suppressing ER stress in ccRCC

**Fig. 5:**
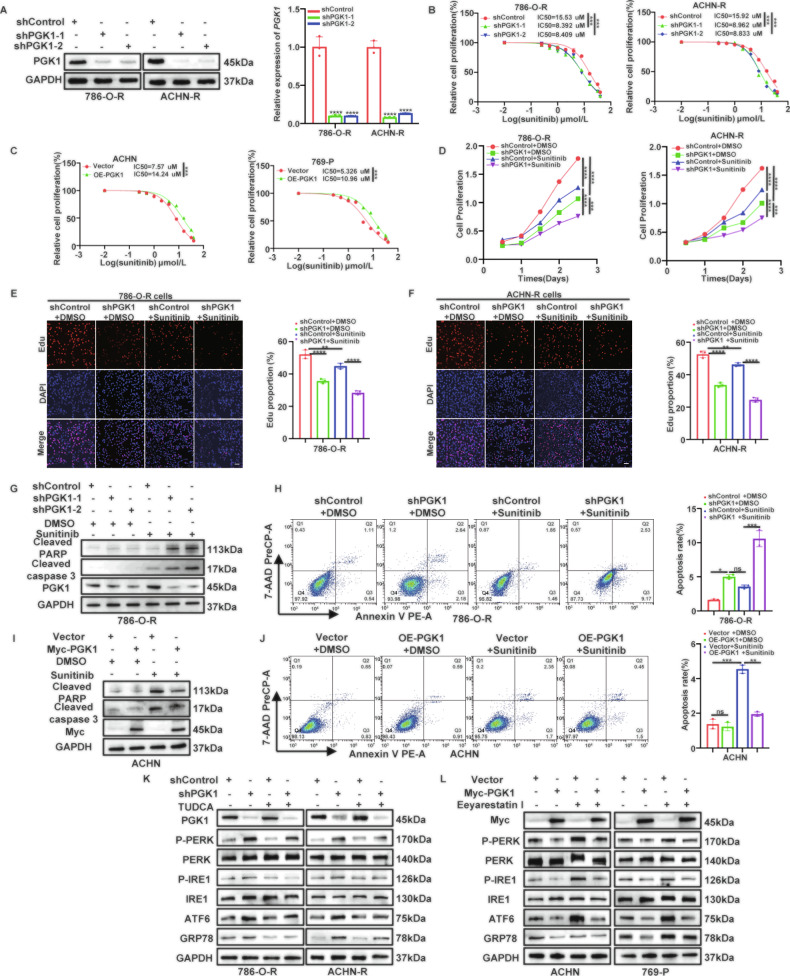
PGK1 contributes to PABPC1-induced sunitinib resistance by suppressing ER stress in ccRCC. **A** qRT-PCR and WB analysis to verify the knockdown efficiency of PGK1 in 786-O-R and ACHN-R cells. **B, C** 786-O-R and ACHN-R cells were infected with lentivirus-expressing shControl or shPGK1 for 72 h, and ACHN and 769-P cells were infected with EV or Myc-PGK1 for 72 h. These cells were treated with a serial dose of sunitinib for 24 h and subjected to CCK-8 assay. Then, the IC50 values were calculated by analysis of the CCK-8 results. **D-F** 786-O-R and ACHN-R cells infected with indicated plasmids were treated with or without 8 µM sunitinib for 24 h. These cells were harvested for CCK-8 (D) (*n* = 3 per group) assays or EdU assays (E, F) (*n* = 3 per group). Scale bar, 100 µm. **G, H** 786-O-R cells infected with indicated plasmids were treated with or without 8 µM sunitinib for 24 h. These cells were harvested for WB analysis **G** or Annexin V‑PE/7‑AAD analysis **H**. **I, J** ACHN cells infected with indicated plasmids were treated with or without 6 µM sunitinib for 24 h. These cells were harvested for WB analysis **I** or Annexin V‑PE/7‑AAD analysis **J**. **K** 786-O-R and ACHN-R cells infected with indicated plasmids were treated with or without 100 μM TUDCA for 24 h. These cells were harvested for WB analysis. **L** ACHN and 769-P cells infected with indicated plasmids were treated with or without 10 µM Eeyarestatin I for 24 h. These cells were harvested for WB analysis (*n* = 3 per group). Data are presented as mean ± SD. Ns, not significant, *P < 0.05, **P < 0.01, ***P < 0.001. P values are calculated by Student’s t test or one-way ANOVA.

Having established PGK1 as a critical downstream effector in PABPC1-mediated ccRCC progression, we further investigated its functional pleiotropy in ccRCC. WB and qRT-PCR confirmed that PGK1 expression was elevated in sunitinib-resistant cell lines (Figure S5A). Similarly, PGK1 protein levels were higher in clinical samples from sunitinib-resistant patients compared to sunitinib-sensitive patients (Figure S5B). Consistent results were observed in various ccRCC cell lines, where PGK1 expression was significantly increased at both the protein and mRNA levels (Figure S5C). Given the elevated PGK1 expression in sunitinib-resistant cell lines and patient samples, as well as its potential regulation by PABPC1, we investigated whether PGK1 mediates PABPC1-induced sunitinib resistance in ccRCC. Initially, we observed that PGK1 knockdown (Fig. 5A) decreased the IC50 values of sunitinib in 786-O-R and ACHN-R cells (Fig. 5B), whereas PGK1 overexpression (Figure S5D) increased sunitinib IC50 values in ACHN and 769-P cells (Fig. 5C). CCK-8 and EdU assays further demonstrated that PGK1 depletion reduced ccRCC cell proliferation and enhanced the antiproliferative effect of sunitinib (Fig. 5D-F). Conversely, PGK1 overexpression significantly promoted ccRCC proliferation even under sunitinib treatment (Figure S5E-G). Furthermore, Annexin V‑PE/7‑AAD staining revealed that PGK1 silencing markedly increased apoptosis in 786-O-R cells, accompanied by increased cleaved caspase-3 and cleaved PARP levels, particularly under combined treatment with sunitinib (Fig. 5G, H). In contrast, PGK1 overexpression inhibited apoptosis induced by sunitinib, as further confirmed by WB analysis (Fig. 5I, J).

Next, we assessed whether PGK1 modulates sunitinib sensitivity via ER stress suppression. ER Tracker imaging revealed significantly enhanced ER staining in PGK1-silenced cells (Figure S5H), whereas ER staining was notably diminished in PGK1-overexpressing cells (Figure S5I). Functional rescue experiments were then performed to elucidate ER stress’s role in PGK1-mediated sunitinib resistance in ccRCC. Treatment of 786-O-R and ACHN-R cells stably silenced for PGK1 with the TUDCA partially reversed the activation of ER stress sensors (Fig. 5K). Moreover, inhibition of ER stress by TUDCA markedly restored proliferative capacity in PGK1-silenced cells (Figure S5J). Conversely, treatment of ACHN and 769-P cells overexpressing PGK1 with Eeyarestatin I reversed the suppression of ER stress sensors caused by PGK1 overexpression (Fig. 5L). Additionally, CCK-8 assays demonstrated that ER stress activation substantially abrogated the enhanced proliferation induced by PGK1 overexpression in ccRCC cells (Figure S5K). Collectively, these findings indicate that PGK1 contributes to PABPC1-induced sunitinib resistance in ccRCC by suppressing ER stress.

### Eeyarestatin I strengthens the antitumor effect of sunitinib in ccRCC

**Fig. 6:**
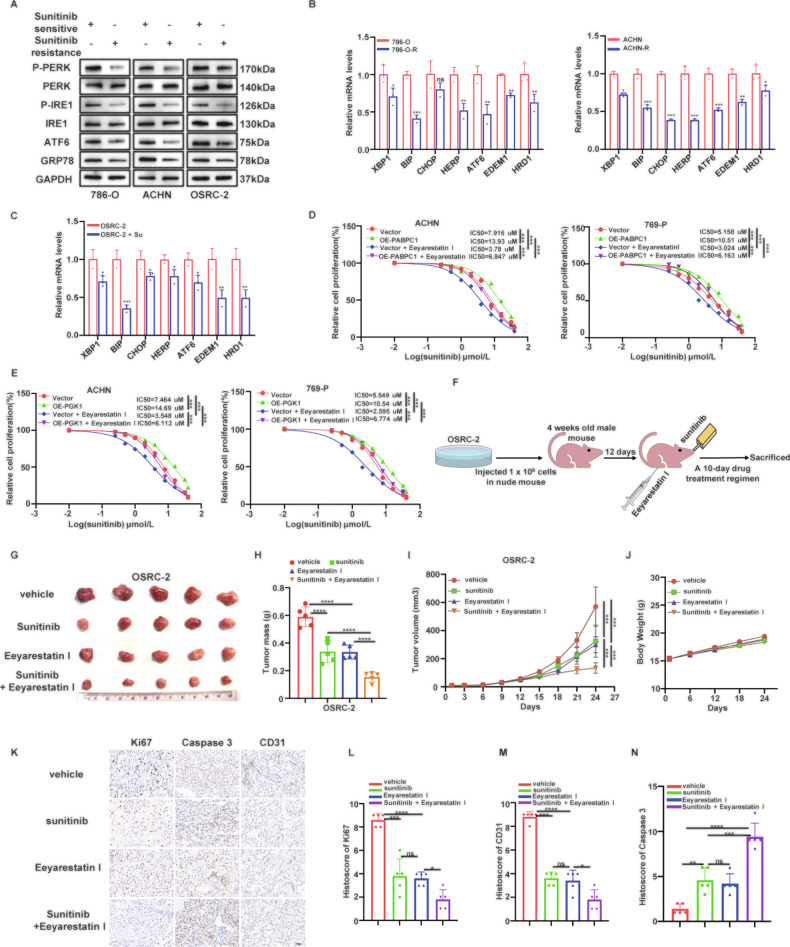
Eeyarestatin I strengthens the antitumor effect of sunitinib in ccRCC. **A–C** qRT-PCR and WB analysis to verify the suppression of sunitinib to ER stress sensors in ccRCC cells. **D, E** ACHN and 769-P cells infected with indicated plasmids were treated with or without 10 µM Eeyarestatin I for 24 h. Next, these cells were treated with a serial dose of sunitinib for 24 h and subjected to CCK-8 assay. The IC50 values were calculated by analysis of the CCK-8 results. **F–J** OSRC-2 cells were subcutaneously injected into nude mice for the xenograft assay and treated with or without sunitinib as well as Eeyarestatin I for 10 days. Schematic of the xenograft model in nude mice was shown in F. The image of tumor was shown in G. Thetumor mass was demonstrated in H. The tumor growth curve was shown in I. The body weight of mice among different groups was shown in **J**. **K–N** Representative images of IHC staining of tumor tissues from mice inoculated with OSRC-2 cells, and treated with or without sunitinib as well as Eeyarestatin I. Scale bar, 20 µm. Data are presented as mean ± SD. Ns, not significant, *P < 0.05, **P < 0.01, ***P < 0.001. P values are calculated by Student’s t test or one-way ANOVA.

Based on the above in vitro experiments, we investigated whether the acquisition of sunitinib resistance is accompanied by the inhibition of ER stress. WB and qRT-PCR analyses demonstrated that the expression of ER stress sensors was significantly suppressed in 786-O-R, ACHN-R, and OSRC-2 cells treated with sunitinib (Fig. 6A–C). Next, IC50 values for sunitinib were determined in ACHN and 769-P cells overexpressing PABPC1 or PGK1 following treatment with Eeyarestatin I. Results revealed that PABPC1 or PGK1 overexpression significantly elevated sunitinib IC50 values, an effect markedly attenuated by Eeyarestatin I treatment (Fig. 6D, E).

**Fig. 7:**
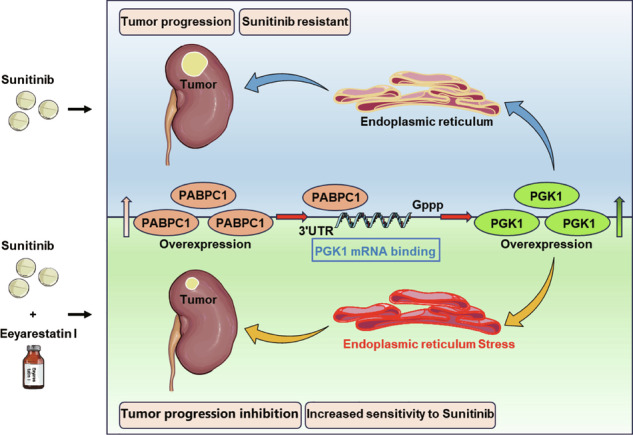
Schematic diagram summarizing the mechanism by which PABPC1 promotes sunitinib resistance in ccRCC. PABPC1 binds to and stabilizes PGK1 mRNA via its 3’UTR, leading to increased PGK1 protein expression. Elevated PGK1 attenuates ER stress, thereby suppressing ER stress-induced apoptosis and conferring resistance to sunitinib, which can be reversed by the small-molecule ER stress inducer, Eeyarestatin I.

Subsequently, we established a xenograft tumor model by subcutaneously injecting OSRC-2 cells into the axillae of nude mice. Combination therapy with Eeyarestatin I significantly enhanced the antitumor effect of sunitinib, as evidenced by notably reduced xenograft tumor volume and weight compared to other treatment groups (Fig. 6F–I). Throughout the treatment period, body weights were monitored weekly as a surrogate for systemic toxicity. No significant differences in body weight were observed among the four groups (Fig. 6J). Moreover, no overt signs of distress, reduced activity, or abnormal behavior were noted in any treatment group. IHC analysis was further performed to evaluate tumor malignancy. The co-treatment group exhibited the lowest expression levels of Ki-67 and CD31 and the highest expression of the apoptosis marker caspase-3 (Fig. 6K-N). Collectively, these findings indicate that sunitinib treatment promotes PABPC1 expression, which enhances PGK1 mRNA stability and expression. Elevated PGK1 levels subsequently inhibit ER stress, reducing ccRCC sensitivity to sunitinib and promoting tumor progression. Crucially, co-treatment with Eeyarestatin I restores sunitinib sensitivity in tumor cells (Fig. 7), representing a promising therapeutic strategy for ccRCC treatment.

## Discussion

In this study, we confirmed that PABPC1 is significantly overexpressed in ccRCC, correlating with poor prognosis and sunitinib resistance. Mechanistically, PABPC1 interacts with the PGK1 3’UTR, protecting it from degradation and consequently enhancing PGK1 expression. Elevated PGK1 levels inhibit ER stress, thereby decreasing ER stress-induced apoptosis and further reducing sunitinib sensitivity in ccRCC. However, treatment with Eeyarestatin I effectively activated ER stress, promoted tumor apoptosis, and restored sunitinib sensitivity. Our findings thus reveal a novel PABPC1-PGK1 regulatory axis underlying sunitinib resistance, highlighting a promising therapeutic strategy to overcome drug resistance in ccRCC.

As a highly conserved RBP in eukaryotes, PABPC1 has been demonstrated to be associated with translation initiation and mRNA stability in various cancers [31–33]. For instance, PABPC1 promotes cell proliferation, migration, invasion, and inhibits apoptosis by interacting with eIF4G to regulate IFI27 mRNA stability and by promoting angiogenesis via exosomal miR-21-5p/CXCL10 signaling in ESCC [34]. Furthermore, previous studies indicated that PABPC1 synergizes with circRNAs to maintain mRNA stability, thereby promoting cisplatin (CDDP) resistance in bladder cancer (BCa) [35]. Wei et al. reported that circSTX6 interacts with PABPC1 to stabilize SUZ12 mRNA, thus reducing the sensitivity of BCa cells to CDDP [36]. Despite extensive evidence highlighting aberrant PABPC1 expression in various cancers, its role in ccRCC, particularly in mediating TKI resistance, remains unclear. In this study, we found that ectopic expression of PABPC1 promoted ccRCC progression and sunitinib resistance both in vitro and in vivo, whereas inhibition of PABPC1 restored sunitinib sensitivity in resistant ccRCC cells. Our findings provide novel evidence that PABPC1 functions as a critical positive regulator of PGK1 at the post-transcriptional level. Specifically, RIP assays confirmed PABPC1 as a PGK1 mRNA-binding protein, while mRNA stability assays demonstrated its role in stabilizing PGK1 mRNA. Elevated PGK1 expression subsequently suppressed ER stress, reducing ER stress-induced apoptosis and decreasing sunitinib sensitivity in ccRCC. Although our data demonstrate that PABPC1 regulates PGK1 primarily through mRNA stabilization, we cannot exclude the possibility that it may also influence translational efficiency. Future studies employing polysome profiling or nascent‑protein labeling could further delineate potential contributions of translational control to this regulatory axis.

PGK1 has multifaceted roles in cancer initiation, tumor progression, and drug resistance [37–39]. The signaling pathways regulated by PGK1 have been extensively studied. For instance, He et al. reported that PGK1-induced metabolic reprogramming activates the CXCR4/ERK signaling pathway, thereby promoting tumor growth and sorafenib resistance in ccRCC [19]. Another study demonstrated that MYC pathway activation in ccRCC promotes cell-cycle progression, proliferation, and anchorage-independent growth by upregulating VEGFA and PGK1 expression [40]. Together, these studies highlight the critical role of PGK1 in ccRCC progression and TKI resistance. Unexpectedly, our RNA-seq data revealed significantly reduced PGK1 expression upon PABPC1 silencing. Subsequent mechanistic experiments further demonstrated that PABPC1 interacts with the 3’UTR of PGK1 mRNA, delaying its degradation and positively regulating its expression. We also confirmed that altering PGK1 expression modulated the IC50 values of sunitinib in resistant ccRCC cells. Moreover, rescue experiments using Eeyarestatin I in PGK1-overexpressing cells reversed the suppression of ER stress sensors, indicating that PGK1 promotes sunitinib resistance by inhibiting ER stress in ccRCC. However, whether PGK1, as a glycolysis-related enzyme, also regulates immune checkpoint inhibition, such as PD-1/PD-L1 blockade, in ccRCC remains to be further clarified.

The ER is an essential organelle with diverse biological functions, including calcium homeostasis, protein synthesis, folding, and post-translational modification [41, 42]. It is highly sensitive to stress, as various factors and conditions can disrupt ER homeostasis and consequently trigger ER stress [43, 44]. Under stress conditions, the UPR, an adaptive signaling pathway, is activated to restore ER equilibrium [45]. Three ER transmembrane proteins function as sensors coordinating the UPR: activating transcription factor 6 (ATF6), inositol-requiring enzyme 1α (IRE1α), and protein kinase R-like ER kinase (PERK) [46–48]. The primary function of ER stress is to restore ER homeostasis; however, prolonged and severe ER stress not only fails to reestablish equilibrium but also induces apoptosis in ccRCC cells, a phenomenon known as ER stress-mediated apoptosis [49, 50]. Increasing evidence indicates that activating ER stress can suppress tumor progression and mitigate drug resistance. Brem et al. demonstrated that combined treatment with bortezomib and Eeyarestatin I significantly enhanced efficacy against cervical cancer cells and reduced bortezomib-related adverse effects, offering insights into individualized cervical cancer treatment strategies [51]. Another study reported that restoring epigenetically silenced PCK2 activated ER stress, suppressing ccRCC progression and enhancing sunitinib sensitivity, thus providing novel therapeutic targets and strategies for ccRCC [52]. However, previous studies have rarely investigated the relationship between PABPC1 and ER stress. In our study, we demonstrated that PABPC1 interacts with PGK1 to suppress ER stress, thereby reducing ER stress-induced apoptosis in ccRCC and ultimately promoting resistance to sunitinib. To our knowledge, this is the first comprehensive report showing that PABPC1 promotes sunitinib resistance by suppressing ER stress, an effect reversible by Eeyarestatin I.

Eeyarestatin I binds directly to the ER membrane-associated p97 ATPase complex, inhibiting substrate deubiquitination and resulting in the accumulation of polyubiquitinated proteins [53]. This process induces ER stress, leading to subsequent tumor cell death. Eeyarestatin I has demonstrated robust antitumor activity against several solid tumors [54]. Co-treatment of Eeyarestatin I and bortezomib markedly enhances the anticancer efficacy against hematological cancer cells, thereby potentially reducing the required bortezomib dosage [44]. In this study, combining Eeyarestatin I with sunitinib significantly enhanced the antitumor efficacy of sunitinib, providing a potential new direction for ccRCC treatment. Targeting PABPC1 or PGK1 may also have therapeutic potential. Unfortunately, inhibitors for PABPC1 or PGK1 remain unavailable. Future research should prioritize designing small-molecule inhibitors targeting PABPC1 or PGK1 for therapeutic use in ccRCC. Furthermore, to better recapitulate the clinical scenario of acquired resistance, future studies will focus on optimizing the in vivo growth conditions of sunitinib‑resistant ccRCC cells to enable direct testing of the PABPC1–PGK1 axis and Eeyarestatin I in a more representative resistant setting.

## Conclusions

In conclusion, our study demonstrates that PABPC1 critically promotes ccRCC progression and resistance to sunitinib. Mechanistically, we provide the first evidence that PABPC1 enhances the mRNA stability of the downstream gene PGK1 by interacting with its 3’UTR. The PABPC1/PGK1 axis promotes ccRCC progression and reduces sunitinib sensitivity through ER stress suppression. However, this resistance can be reversed by Eeyarestatin I, highlighting its potential as a therapeutic strategy for ccRCC treatment.

## Supplementary information

Supplementary figure legends

Figure S1

Figure S2

Figure S3

Figure S4

Figure S5

Supplemental tables

Original western blots

## Data Availability

All data generated or analyzed during this study are included in this published article and its Additional Files. RNA-seq data reported in this paper were deposited in the Gene Expression Omnibus database (GEO GSE314693).
