# Supplementary material for: PABPC1-induced stabilization of PGK1 mRNA reduces apoptosis and sunitinib sensitivity in renal cell carcinoma by suppressing endoplasmic reticulum stress
Source: Cell Death Dis. 2026 Apr 3;17(1):452. doi: 10.1038/s41419-026-08676-3 (PMC13172027; doi:10.1038/s41419-026-08676-3)
Supplement: Supplementary file 1 — Supplementary figure legends [file 41419_2026_8676_MOESM1_ESM.docx]

**Supplementary figure legends**

**Figure S1. Elevated PABPC1 expression is associated with poor prognosis and ccRCC progression. A** Expression of PABPC1 in the immortalized normal cell line HK2 and ccRCC cell lines (OSRC-2, 786-O, ACHN and 769-P) was detected by qRT-PCR and WB. **B, C** qRT-PCR and WB analysis to verify the knockdown efficiency of PABPC1 in 786-O, OSRC-2 cells (B) and overexpression efficiency in ACHN and 769-P cells (C). **D** CCK-8 assays of shControl or shPABPC1 in 786-O and OSRC-2 cells (n = 3 per group). **E** CCK-8 assays of vector or PABPC1-overexpressing in ACHN and 769-P cells (n = 3 per group). **F, H, J, L** Transwell assays (F, H) and Wound healing (J, L) of shControl or shPABPC1 in 786-O and OSRC-2 cells (n = 3 per group). Scale bar, 100 µm. **G, I, K, M** Transwell assays (G, I) and Wound healing (K, M) of vector or PABPC1-overexpressing in ACHN and 769-P cells (n = 3 per group). Scale bar, 100 µm. *P<0.05, **P<0.01, ***P<0.001. P values are calculated by Student’s t test or one-way ANOVA.

**Figure S2. PABPC1 reduces sunitinib sensitivity in ccRCC cells** **in vitro and in vivo.** **A** The protein expression level of PABPC1 from OSRC-2 cells with or without sunitinib treatment was detected by WB. **B** WB analysis of PABPC1 expression in sunitinib sensitive (n=6) and resistance (n=6) ccRCC patients. The expression level of protein was quantified by ImageJ software. **C, D** Scatter plot of the relationship among PABPC1 and the VEGF family. **E** The resistant lines and their corresponding parental cells were treated with a serial dose of sunitinib for 24 h and subjected to CCK-8 assay. Then, the IC50 values were calculated by analysis of the CCK-8 results. **F, G** qRT-PCR and WB analysis to verify the knockdown efficiency of PABPC1 in 786-O-R and ACHN-R cells. **H** 786-O R, ACHN-R and OSRC-2 cells infected with indicated plasmids were treated with a serial dose of sunitinib for 24 h and subjected to CCK-8 assay. Then, the IC50 values were calculated by analysis of the CCK-8 results. **I** ACHN and 769-P cells infected with indicated plasmids were treated with a serial dose of sunitinib for 24 h and subjected to CCK-8 assay. Then, the IC50 values were calculated by analysis of the CCK-8 results. **J, K** 786-O-R, ACHN-R and OSRC-2 cells infected with indicated plasmids were treated with or without sunitinib for 24 h. These cells were harvested for CCK-8 (J) (n = 3 per group) assays or EdU assays (K) (n = 3 per group). Scale bar, 100 µm. **L-N** ACHN and 769-P cells infected with indicated plasmids were treated with or without 6 µM sunitinib for 24 h. These cells were harvested for CCK-8(L) (n = 3 per group) assays or EdU assays (M, N) (n = 3 per group). Data are presented as mean ± SD. *P<0.05, **P<0.01, ***P<0.001. P values are calculated by Student’s t test or one-way ANOVA.

**Figure S3. PABPC1 suppresses ER stress and reduces sunitinib sensitivity in ccRCC. A** Immunofluorescence technology traced ER in ccRCC cells overexpressing PABPC1, Red: ER-tracker, Blue: Hoechst. Scale bar, 10 µm. **B** mRNA levels of ER stress sensors in OSRC-2 cell lines depleting PABPC1.**C** 786-O-R and ACHN-R cells infected with indicated plasmids were treated with or without 100 μM TUDCA for 24 h. These cells were harvested for CCK-8 (n = 3 per group) assays. **D** ACHN and 769-P cells infected with indicated plasmids were treated with or without 10 µM Eeyarestatin I for 24 h. These cells were harvested for CCK-8 (n = 3 per group) assays. Data are presented as mean ± SD. Ns, not significant, *P<0.05, **P<0.01, ***P<0.001. P values are calculated by Student’s t test or one-way ANOVA.

**Figure S4. PGK1 is a critical factor in PABPC1‑mediated ccRCC progression. A** Comparative TCGA analysis identified six tumor‑upregulated genes among 12 high‑confidence candidates. The expression levels of CCM2L, CLDN2, LTB, PGK1, TNFSF14 and VCAM1 were significantly higher in ccRCC tumors than in adjacent normal tissues. **B** Detection of six candidate mRNA levels by qRT-PCR upon PABPC1 silencing in 786-O and OSRC-2 cells (n = 3). **C** Detection of PGK1 pre-mRNA levels by qRT-PCR in 786-O and OSRC-2 cell lines silencing PABPC1, as well as ACHN and 769-P cell lines overexpressing PABPC1. **D** ACHN and 769-P cell lines overexpressing PABPC1 were treated with actinomycin D (5 mg/mL) to quantify PGK1 mRNA levels by qRT-PCR. **E-G** 786-O and OSRC-2 cells infected with indicated plasmids were harvested for CCK-8 assays (E) (n = 3 per group) and EdU assays (F, G) (n = 3 per group). Scale bars: 100 µm. **H-J** 786-O and OSRC-2 cells infected with indicated plasmids were harvested for Transwell assays (n = 5 per group). Scale bars: 100 µm. Data are presented as mean ± SD. *P<0.05, **P<0.01, ***P<0.001. P values are calculated by Student’s t test or one-way ANOVA.

**Figure S5. PGK1 contributes to** **PABPC1-induced sunitinib resistance by suppressing ER stress in ccRCC. A** The protein and mRNA expression level of PGK1 from 786-O, ACHN and OSRC-2 cells with or without sunitinib resistance was detected by WB and qRT-PCR. **B** WB analysis of PGK1 expression in sunitinib sensitive (n=6) and resistance (n=6) ccRCC patients. The expression level of protein was quantified by ImageJ software. **C** Expression of PGK1 in the immortalized normal cell line HK2 and ccRCC cell lines (OSRC-2, 786-O, ACHN,769-P) was detected by qRT-PCR and WB. **D** qRT-PCR and WB to verify the overexpression efficiency of PGK1 in ACHN and 769-P cells. **E-G** ACHN and 769-P cells infected with indicated plasmids were treated with or without 6 µM sunitinib for 24 h. These cells were harvested for EdU assays (E, F) (n = 3 per group) or CCK-8 assays (G) (n = 3 per group). Scale bars: 100 µm. **H, I** Immunofluorescence technology traced ER in ccRCC cells depleting or overexpressing PGK1, Red: ER-tracker, Blue: Hoechst. Scale bar, 10 µm. **J** 786-O-R and ACHN-R cells infected with indicated plasmids were treated with or without 100 μM TUDCA for 24 h. These cells were harvested for CCK-8 assays (n = 3 per group). **K** ACHN and 769-P cells infected with indicated plasmids were treated with or without 10 µM Eeyarestatin I for 24 h. These cells were harvested for CCK-8 assays (n = 3 per group). Data are presented as mean ± SD. Ns, not significant, *P<0.05, **P<0.01, ***P<0.001. P values are calculated by Student’s t test or one-way ANOVA.
