# Supplementary figures and images for: PABPC1-induced stabilization of PGK1 mRNA reduces apoptosis and sunitinib sensitivity in renal cell carcinoma by suppressing endoplasmic reticulum stress
Source: Cell Death Dis. 2026 Apr 3;17(1):452. doi: 10.1038/s41419-026-08676-3 (PMC13172027; doi:10.1038/s41419-026-08676-3)

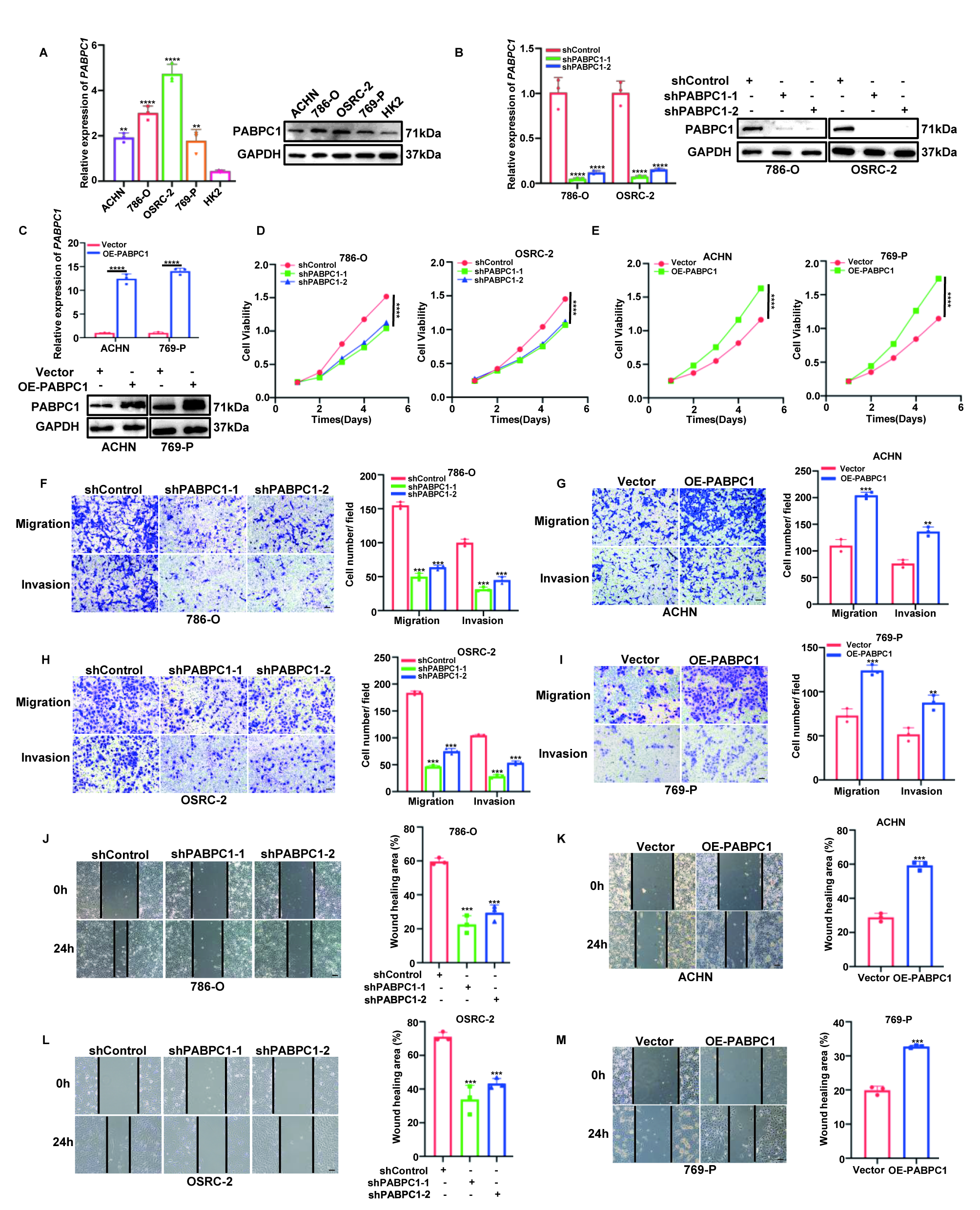

Supplement: Supplementary file 2 — Figure S1 [file 41419_2026_8676_MOESM2_ESM.tif]

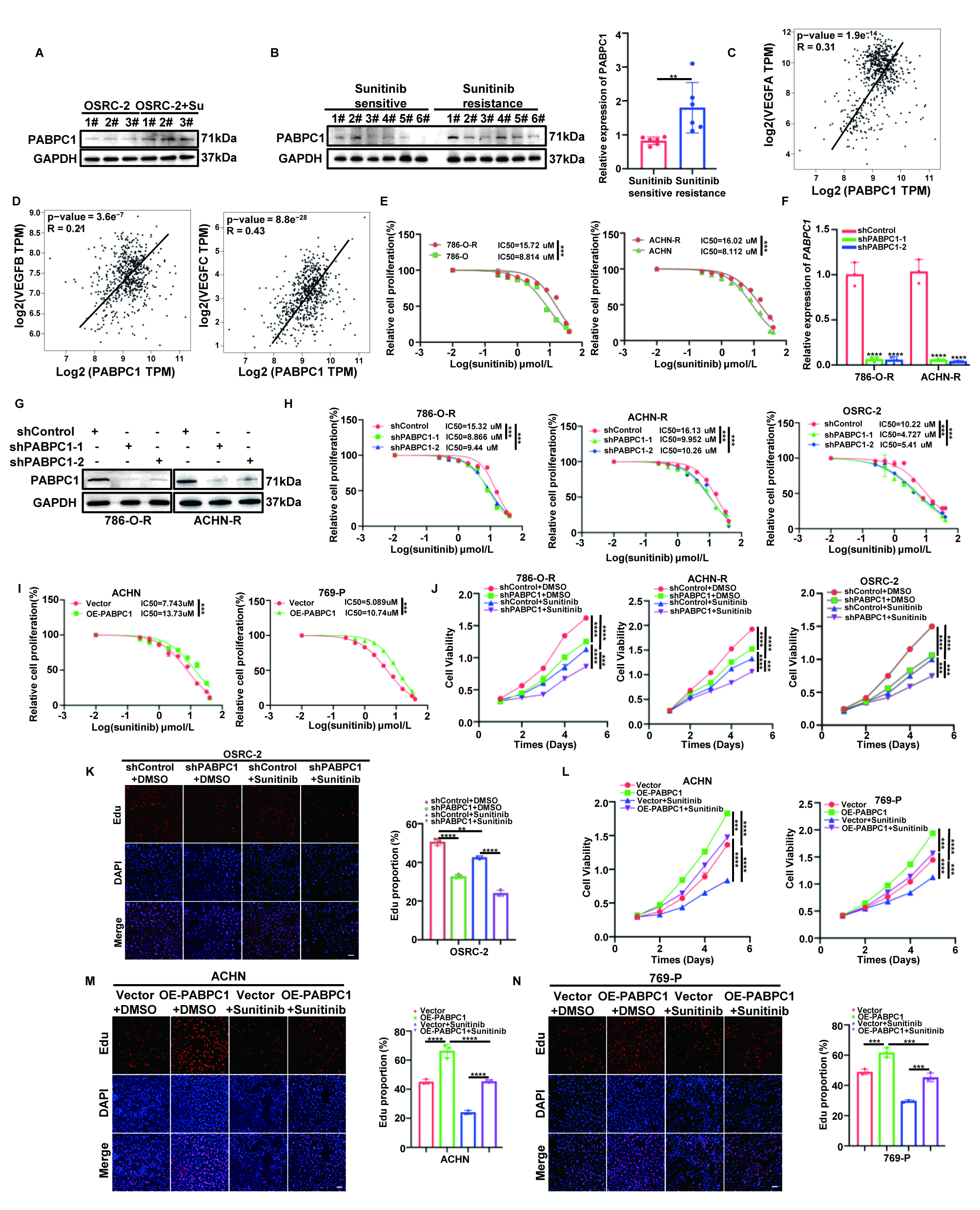

Supplement: Supplementary file 3 — Figure S2 [file 41419_2026_8676_MOESM3_ESM.tif]

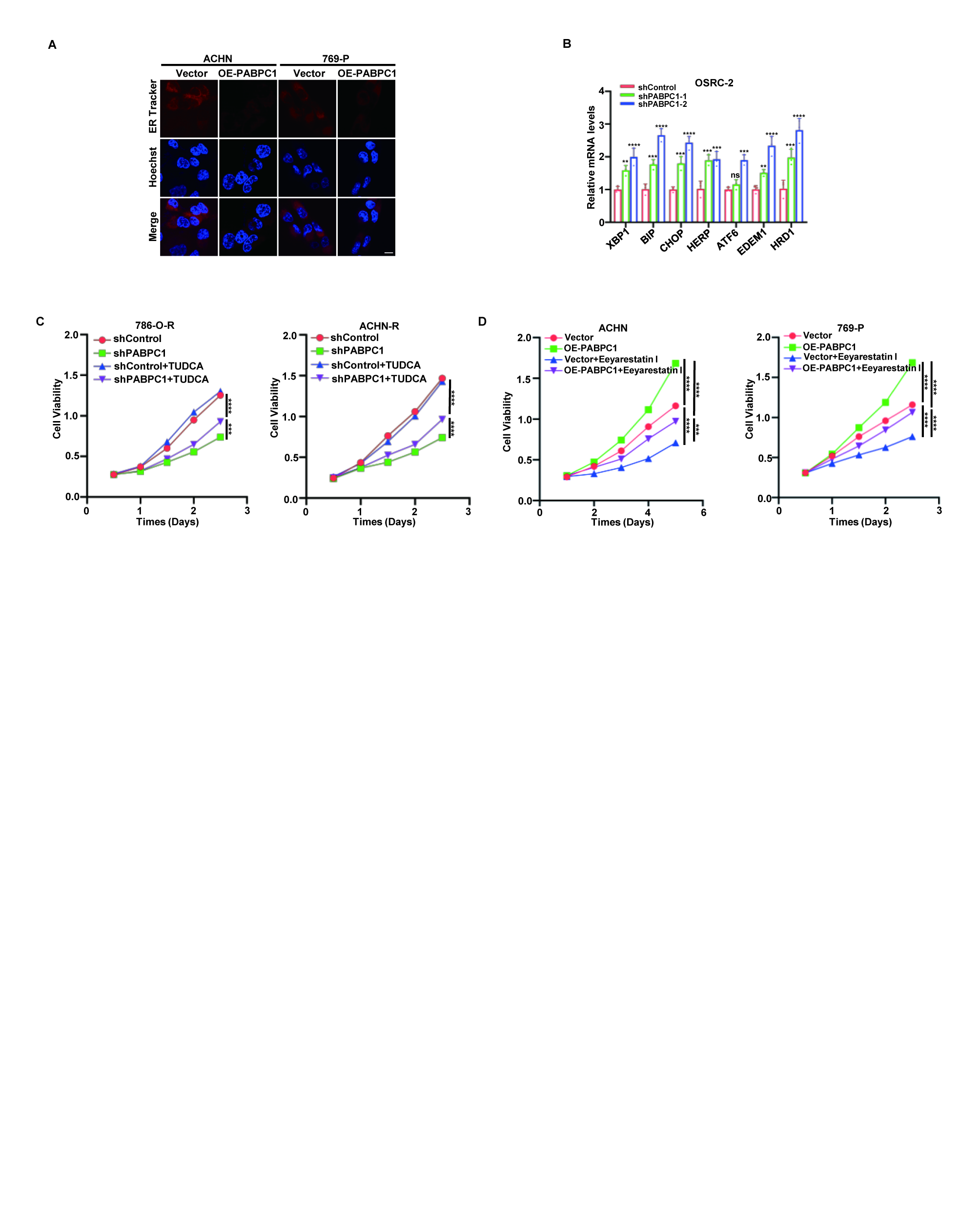

Supplement: Supplementary file 4 — Figure S3 [file 41419_2026_8676_MOESM4_ESM.tif]

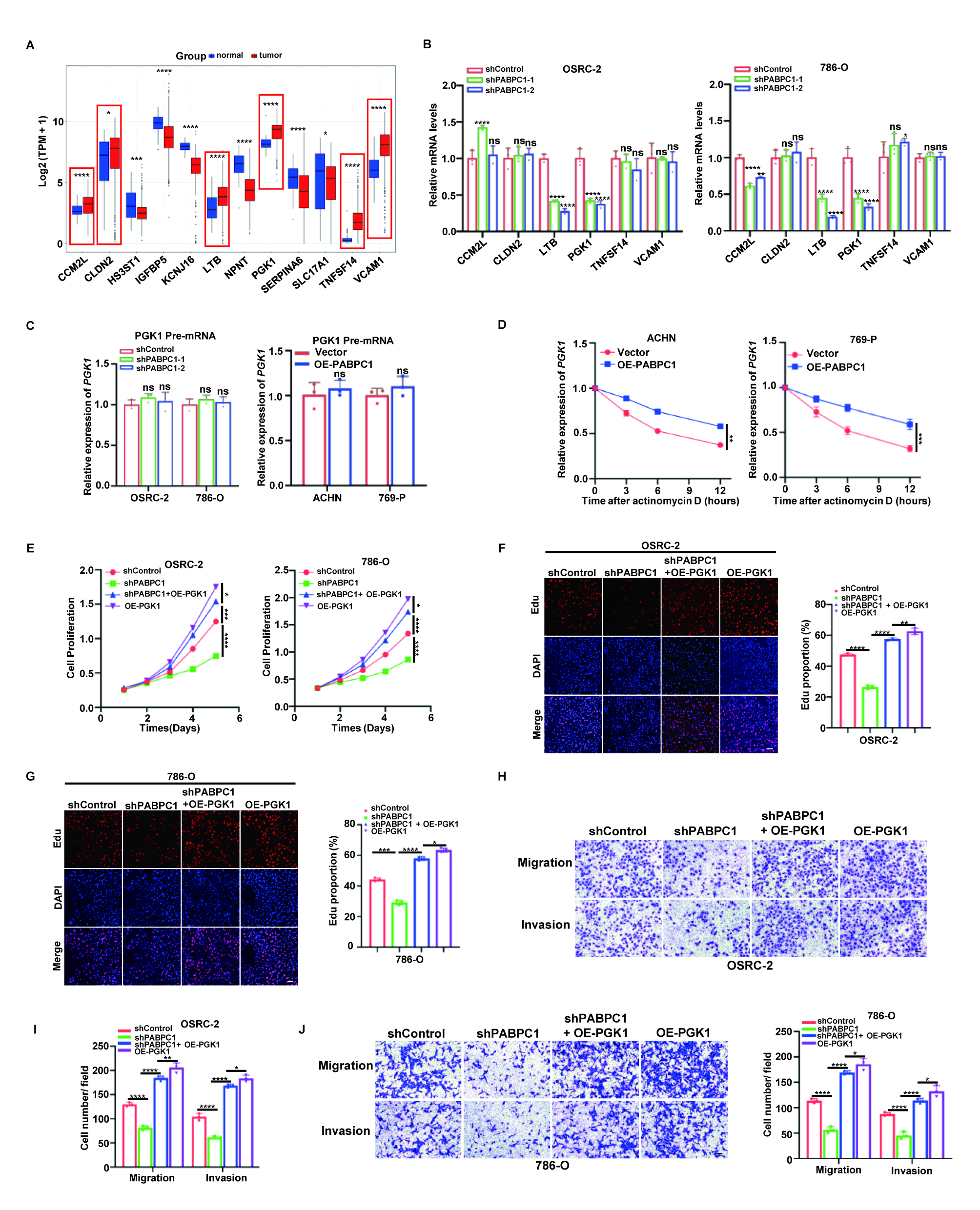

Supplement: Supplementary file 5 — Figure S4 [file 41419_2026_8676_MOESM5_ESM.tif]

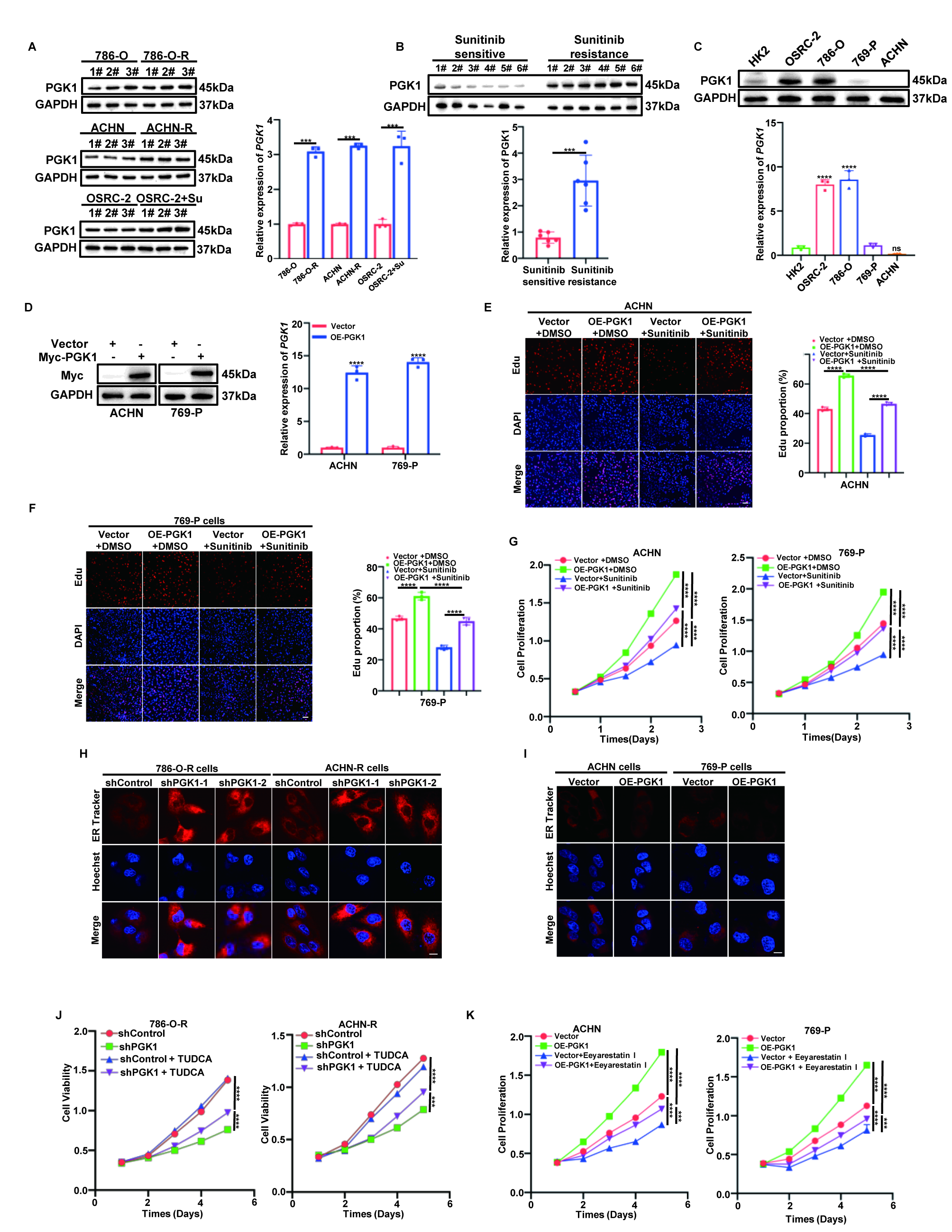

Supplement: Supplementary file 6 — Figure S5 [file 41419_2026_8676_MOESM6_ESM.tif]
