# Supplementary material for: PABPC1-induced stabilization of PGK1 mRNA reduces apoptosis and sunitinib sensitivity in renal cell carcinoma by suppressing endoplasmic reticulum stress
Source: Cell Death Dis. 2026 Apr 3;17(1):452. doi: 10.1038/s41419-026-08676-3 (PMC13172027; doi:10.1038/s41419-026-08676-3)
Supplement: Supplementary file 7 — Supplemental tables [file 41419_2026_8676_MOESM7_ESM.docx]

| **Supplemental Table1. ShRNA oligo sequences.** | | |
| --- | --- | --- |
| **Vector** | **Oligonucleotide** | **Sequence (5′-3′)** |
| shPABPC1#1 | Forward | CCGGGCCGCACCGTTCCACAGTATAACTCGAGTTATACTGTGGAACGGTGCGGTTTTTG |
|  | Reverse | AATTCAAAAACCGCACCGTTCCACAGTATAACTCGAGTTATACTGTGGAACGGTGCGGC |
| shPABPC1#2 | Forward | CCGGGCCAGACCTCATCCATTCCAAACTCGAGTTTGGAATGGATGAGGTCTGGTTTTTG |
|  | Reverse | AATTCAAAAACCAGACCTCATCCATTCCAAACTCGAGTTTGGAATGGATGAGGTCTGGC |
| shPGK1#1 | Forward | CCGGGCCAAGATTGTCAAAGACCTACTCGAGTAGGTCTTTGACAATCTTGGCTTTTTG |
|  | Reverse | AATTCAAAAAGCCAAGATTGTCAAAGACCTACTCGAGTAGGTCTTTGACAATCTTGGC |
| shPGK1#2 | Forward | CCGGCTGACAAGTTTGATGAGAATGCTCGAGCATTCTCATCAAACTTGTCAGTTTTTG |
|  | Reverse | AATTCAAAAACTGACAAGTTTGATGAGAATGCTCGAGCATTCTCATCAAACTTGTCAG |

| **Supplemental Table2. The sequences of primers for qRT-PCR.** | | |
| --- | --- | --- |
| **Gene** | **Primer** | **Sequence (5′-3′)** |
| PABPC1 | Forward | GCCAGACCTCATCCATTCCAA |
|  | Reverse | ACGCTGTGTTGACATGACTCG |
| PGK1 | Forward | GAACAAGGTTAAAGCCGAGCC |
|  | Reverse | GTGGCAGATTGACTCCTACCA |
| XBP1 | Forward | AGCTCAGACTGCCAGAGATC |
|  | Reverse | TCACTTCATTCCCCTTGGCT |
| BIP | Forward | AGGACAAGAAGGAGGACGTG |
|  | Reverse | ATCAGACGTTCCCCTTCAGG |
| CHOP | Forward | CATTGCCTTTCTCCTTCGGG |
|  | Reverse | CCAGAGAAGCAGGGTCAAGA |
| HERP | Forward | GAAAACCAGCCTGCCAATCA |
|  | Reverse | GTACATAACAACGGTGGCCC |
| ATF6 | Forward | GTGTCAGAGAACCAGAGGCT |
|  | Reverse | GGTGCCTCCTTTGATTTGCA |
| EDEM1 | Forward | TGGAAACGATATGGTGCCCT |
|  | Reverse | TCTCCATCCGGTCTTCTGTG |
| HRD1 | Forward | ATCCTGATGACGATGGTGCT |
|  | Reverse | TGAAGGCCATGTACAGCAGA |
| VCAM1 | Forward | GGGAAGATGGTCGTGATCCTT |
|  | Reverse | TCTGGGGTGGTCTCGATTTTA |
| TNFSF14 | Forward | ATACAAGAGCGAAGGTCTCACG |
|  | Reverse | CTGAGTCTCCCATAACAGCGG |
| LTB | Forward | GTACGGGCCTCTCTGGTACA |
|  | Reverse | GTCCACCATATCGGGGTGAC |
| CCM2L | Forward | CCTGCAACCTGGTCATCCTGGCTGT |
|  | Reverse | TACTCCCGCAGCAGCATCGCAA |
| CLDN2 | Forward | GCCTCTGGATGGAATGTGCC |
|  | Reverse | GCTACCGCCACTCTGTCTTTG |
| RIP | Forward | TTCTGCATCTCCACTTGGCAT |
|  | Reverse | GAACTAAGCTAACACTGCTCAC |
| PPIA | Forward | ATGGTCAACCCCACCGTGT |
|  | Reverse | TCTGCTGTCTTTGGGACCTTGTC |
