# Supplementary material for: PABPC1-induced stabilization of PGK1 mRNA reduces apoptosis and sunitinib sensitivity in renal cell carcinoma by suppressing endoplasmic reticulum stress
Source: Cell Death Dis. 2026 Apr 3;17(1):452. doi: 10.1038/s41419-026-08676-3 (PMC13172027; doi:10.1038/s41419-026-08676-3)

Fig. 1E

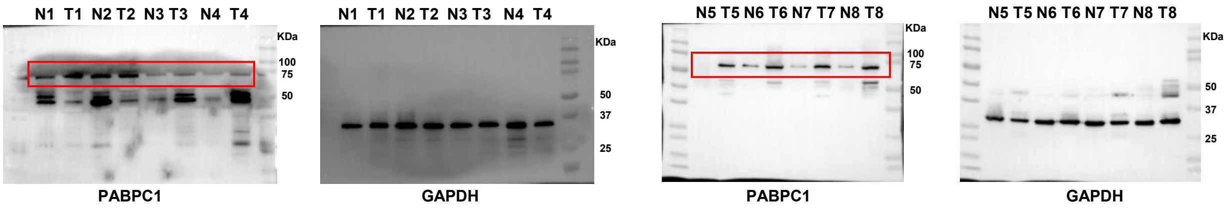

Fig. 2A

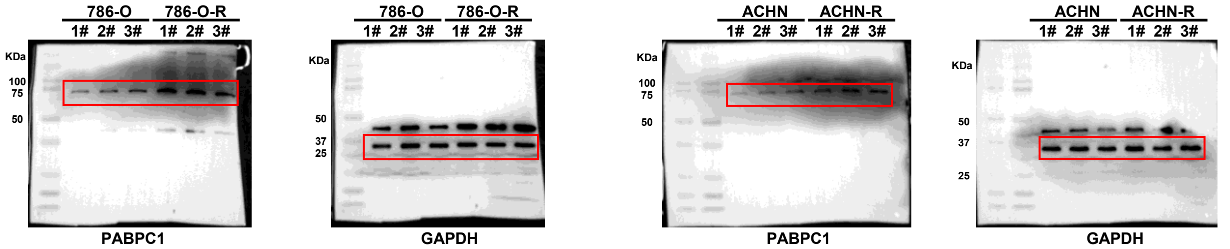

Fig. 2E

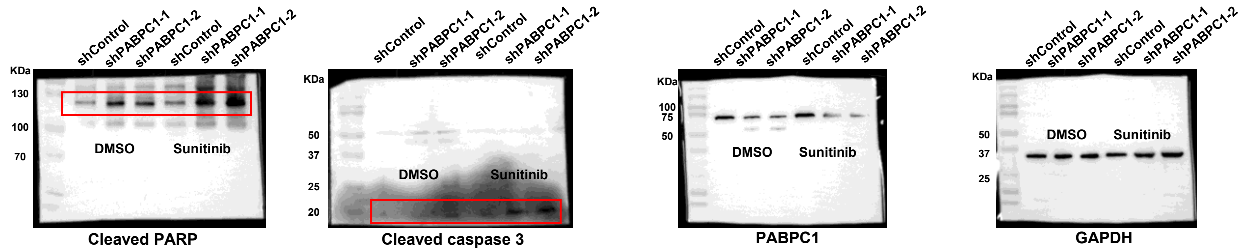

Fig. 2H

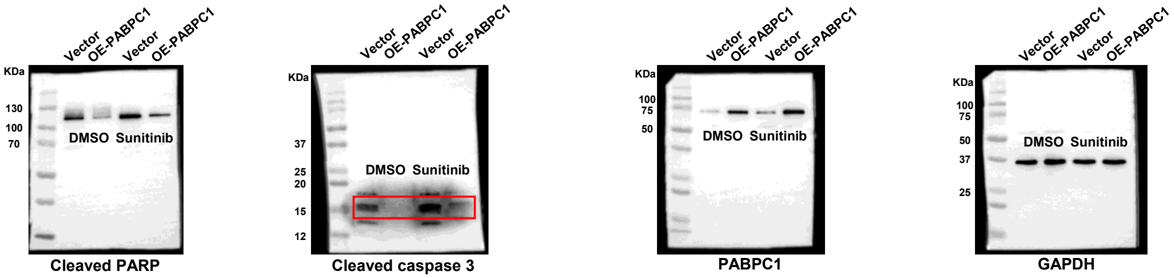

Fig. 3F

786-O-R

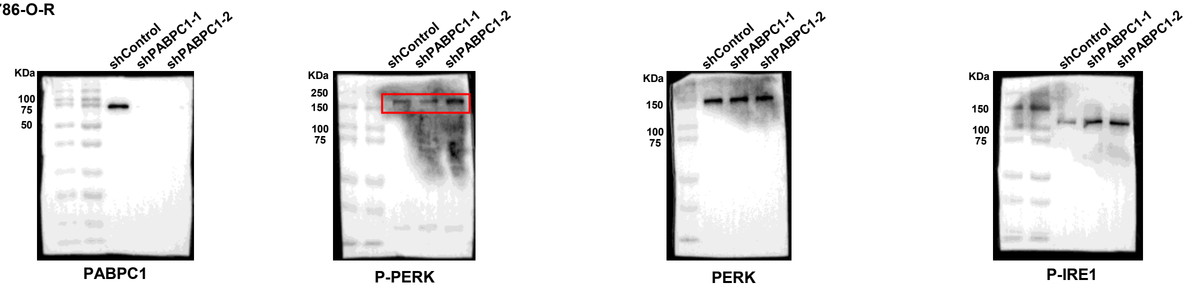

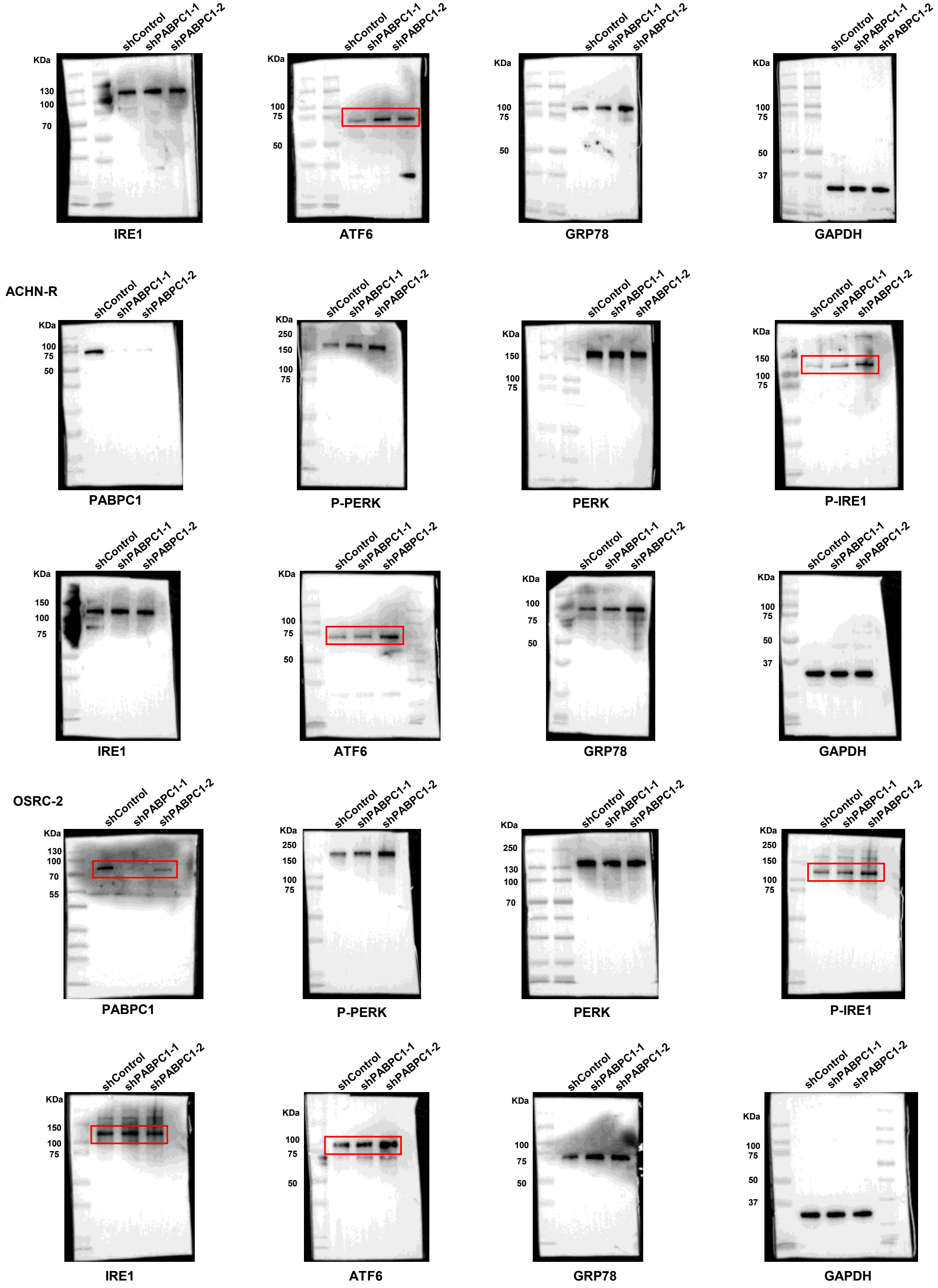

ACHN-R
OSRC-2
KDa
shControl
shPABPC1-1
shPABPC1-2
IRE1
ATF6
GRP78
GAPDH
PABPC1
P-PERK
PERK
P-IRE1
250
150
130
100
75
70
55
50
37

Fig. 3H

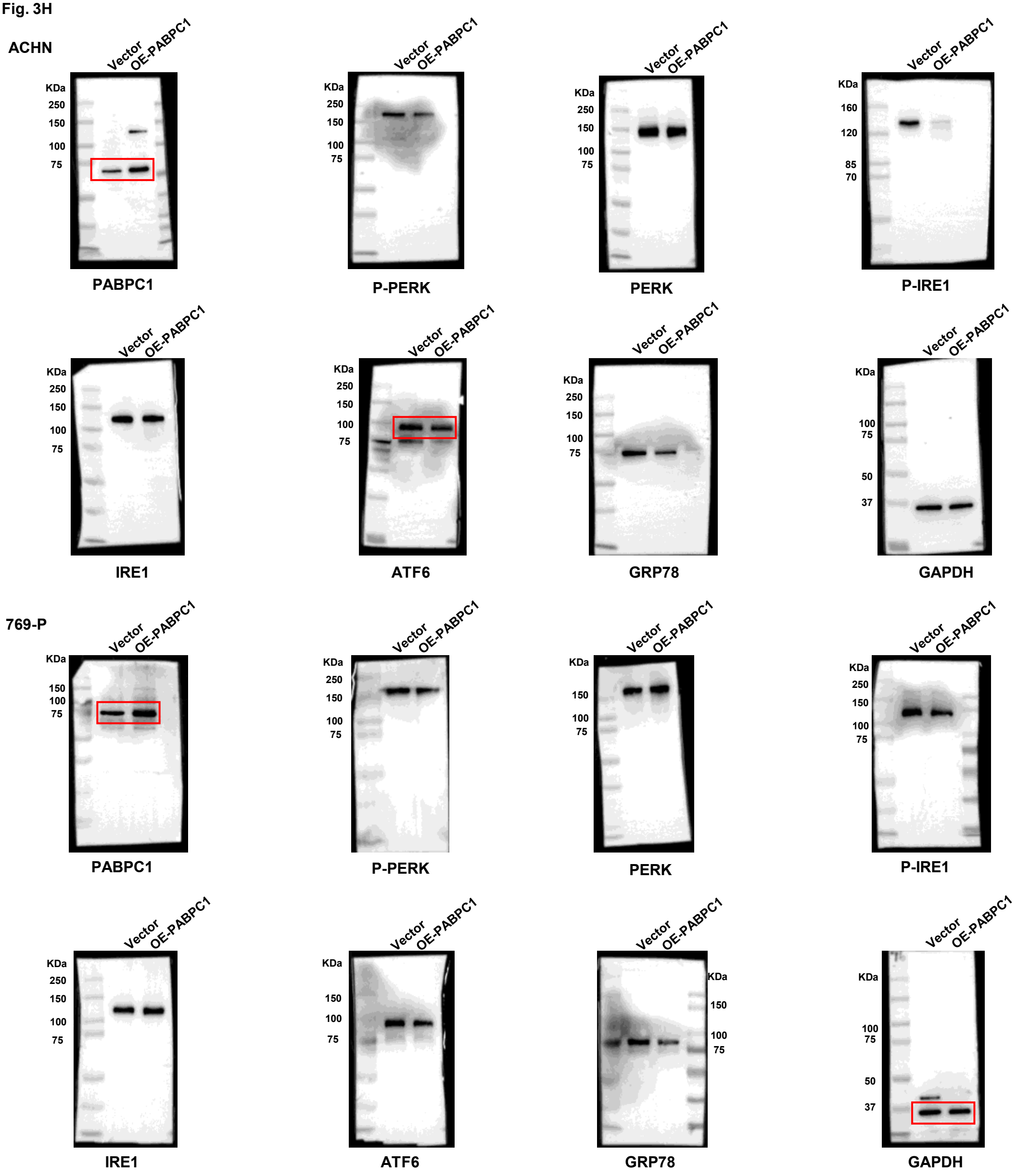
ACHN
Vector
OE-PABPC1
PABPC1
P-PERK
PERK
P-IRE1
IRE1
ATF6
GRP78
GAPDH
769-P
Vector
OE-PABPC1
PABPC1
P-PERK
PERK
P-IRE1
IRE1
ATF6
GRP78
GAPDH

Fig. 3J

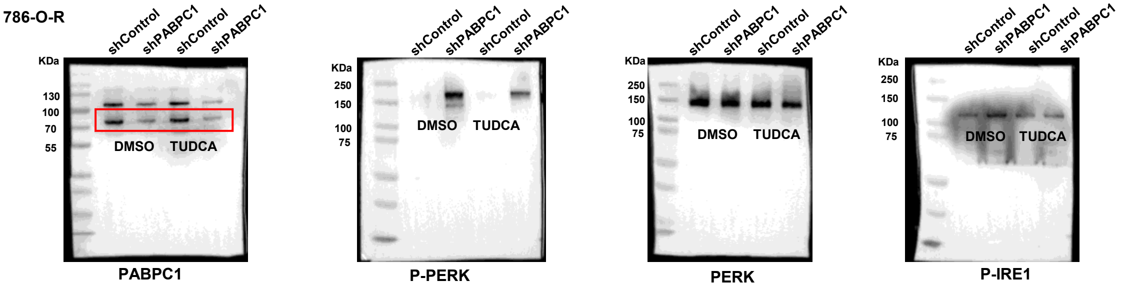
786-O-R
shControl
shPABPC1
shControl
shPABPC1
DMSO
TUDCA
PABPC1
P-PERK
PERK
P-IRE1

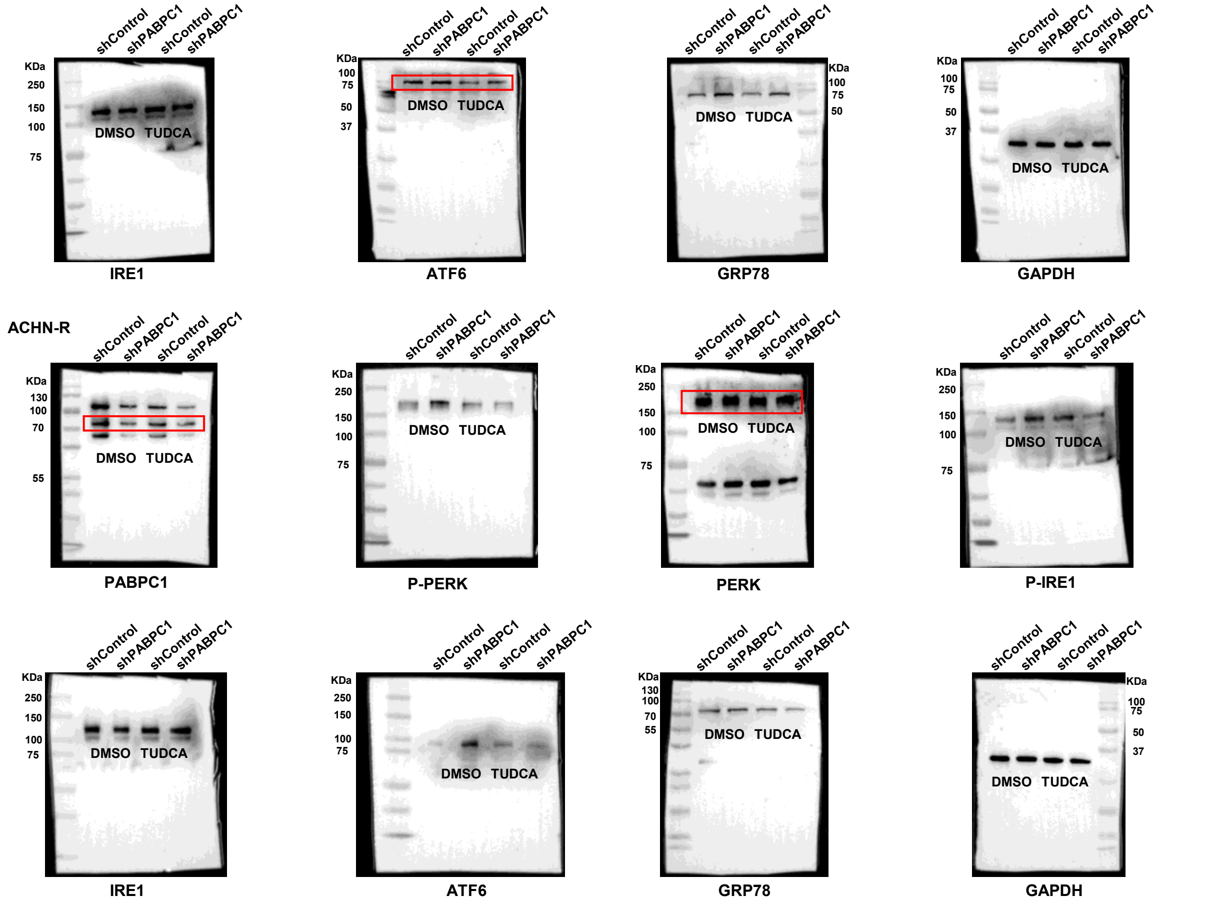
ACHN-R
shControl
shPABPC1
shControl
shPABPC1
KDa
DMSO
TUDCA
IRE1
ATF6
GRP78
GAPDH
PABPC1
P-PERK
PERK
P-IRE1
IRE1
ATF6
GRP78
GAPDH

Fig. 3K

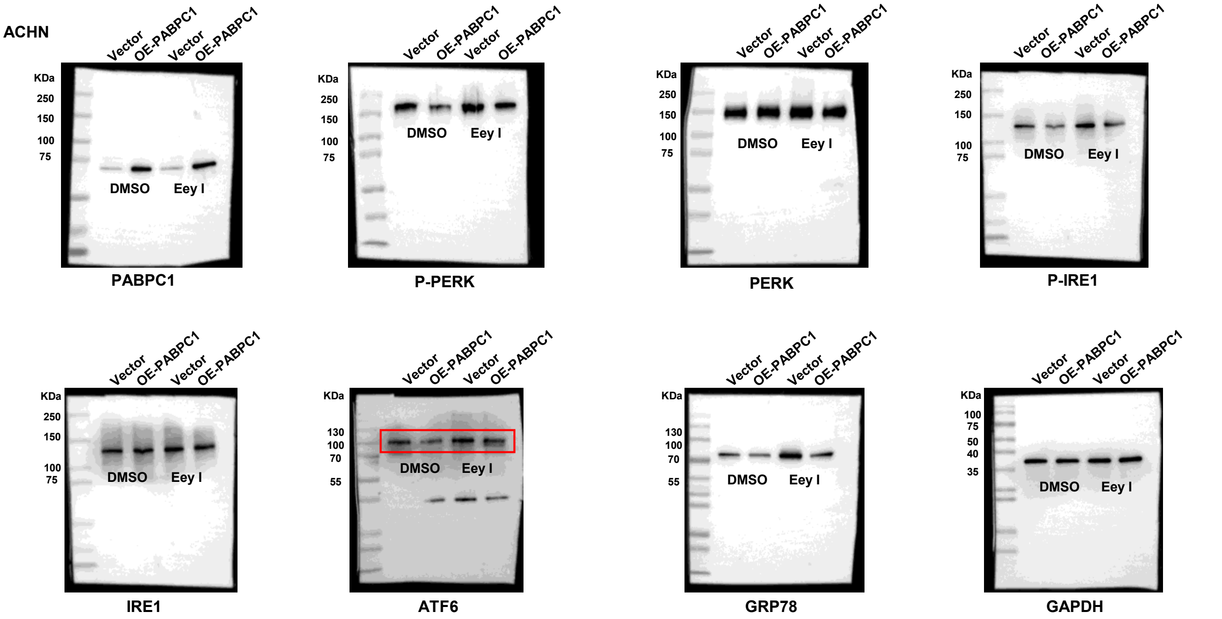
ACHN
Vector
OE-PABPC1
Vector
OE-PABPC1
KDa
DMSO
Eey I
PABPC1
P-PERK
PERK
P-IRE1
IRE1
ATF6
GRP78
GAPDH

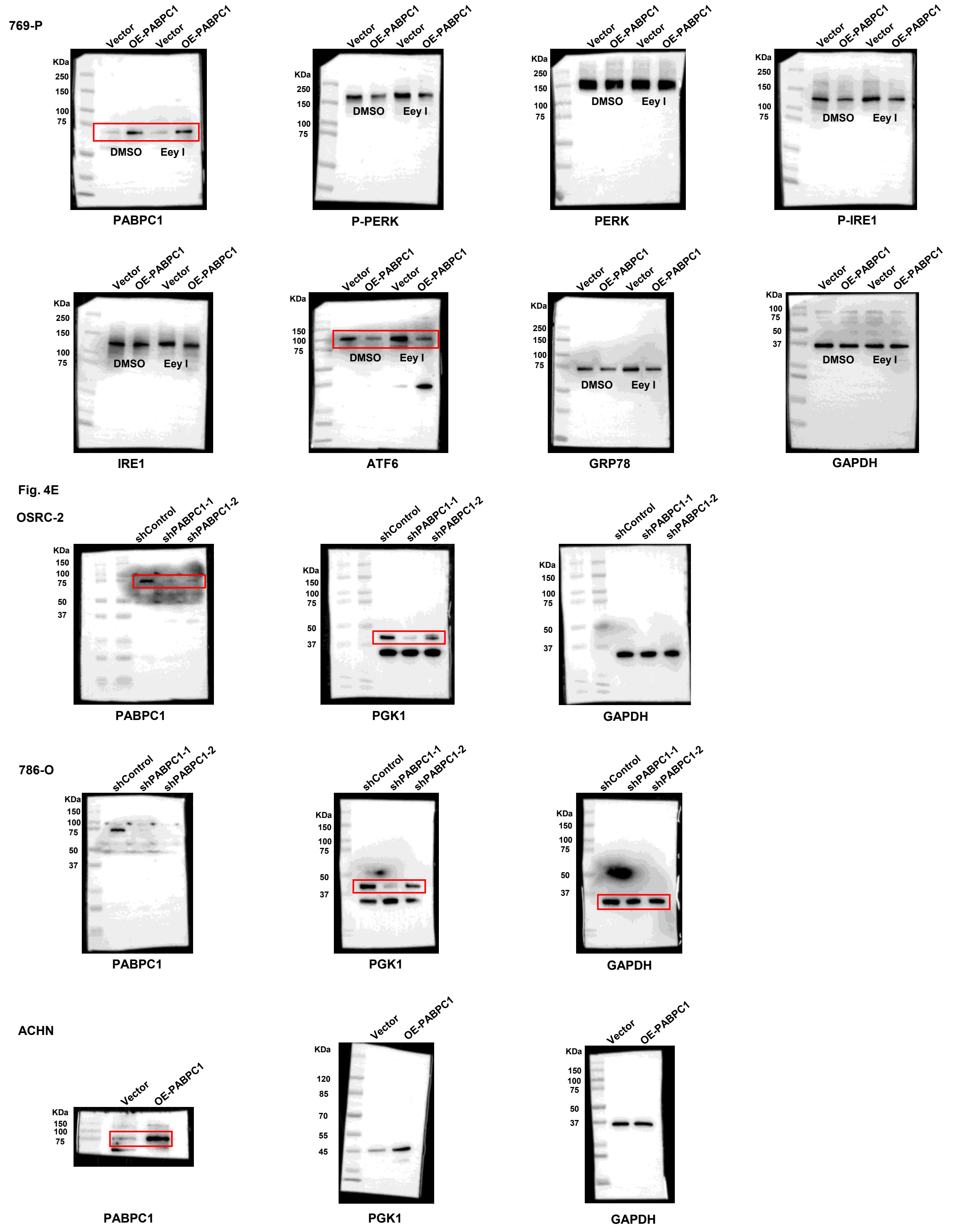

769-P
Vector
OE-PABPC1
Vector
OE-PABPC1
KDa
DMSO
Eey I
PABPC1
P-PERK
PERK
P-IRE1
IRE1
ATF6
GRP78
GAPDH
Fig. 4E
OSRC-2
shControl
shPABPC1-1
shPABPC1-2
PABPC1
PGK1
GAPDH
786-O
shControl
shPABPC1-1
shPABPC1-2
PABPC1
PGK1
GAPDH
ACHN
Vector
OE-PABPC1
PABPC1
PGK1
GAPDH

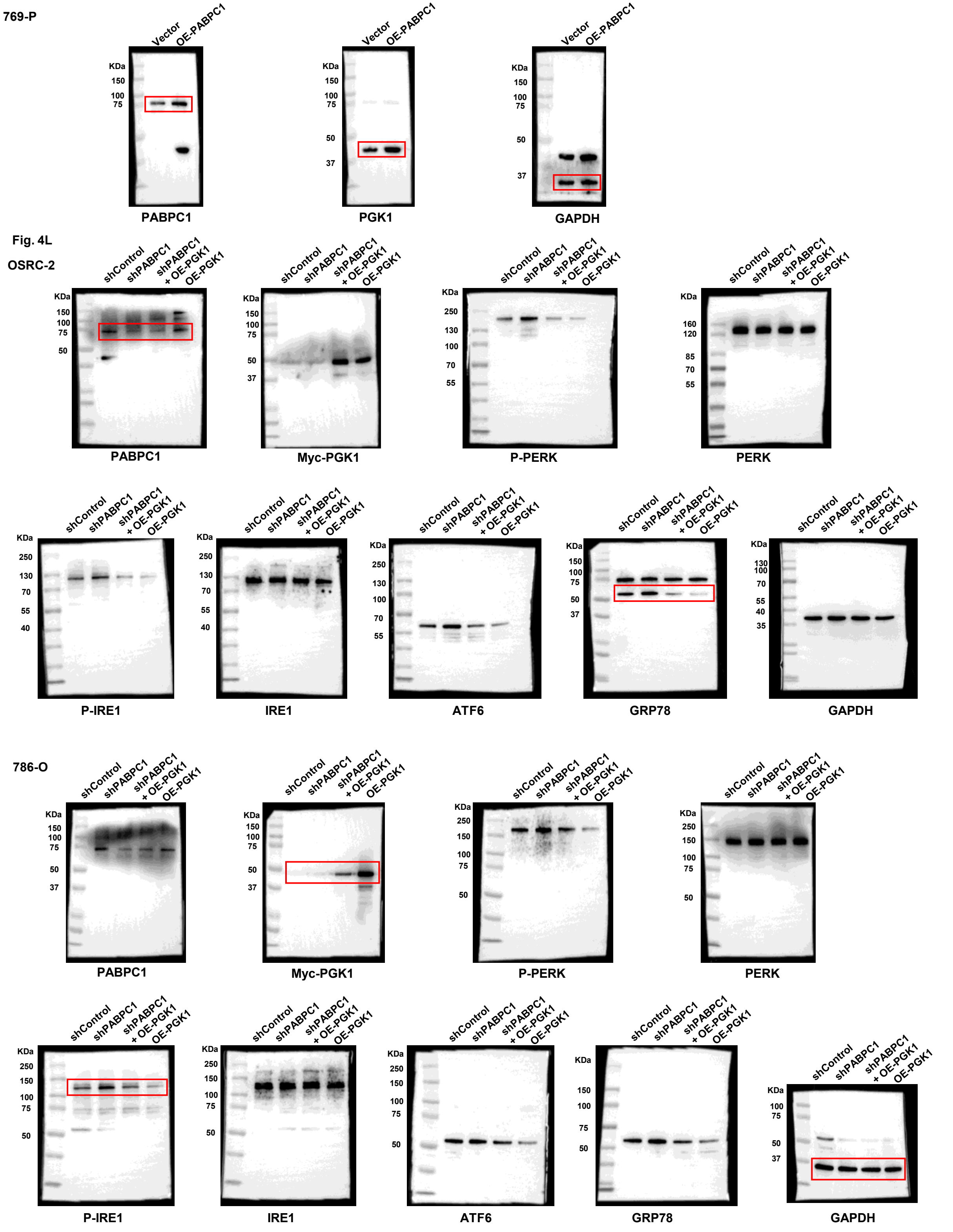

769-P
Vector
OE-PABPC1
KDa
150
100
75
50
37
PABPC1
PGK1
GAPDH
Fig. 4L
OSRC-2
shControl
shPABPC1
shPABPC1 + OE-PGK1
OE-PGK1
PABPC1
Myc-PGK1
P-PERK
PERK
P-IRE1
IRE1
ATF6
GRP78
GAPDH
786-O
shControl
shPABPC1
shPABPC1 + OE-PGK1
OE-PGK1
PABPC1
Myc-PGK1
P-PERK
PERK
P-IRE1
IRE1
ATF6
GRP78
GAPDH

Fig. 5A

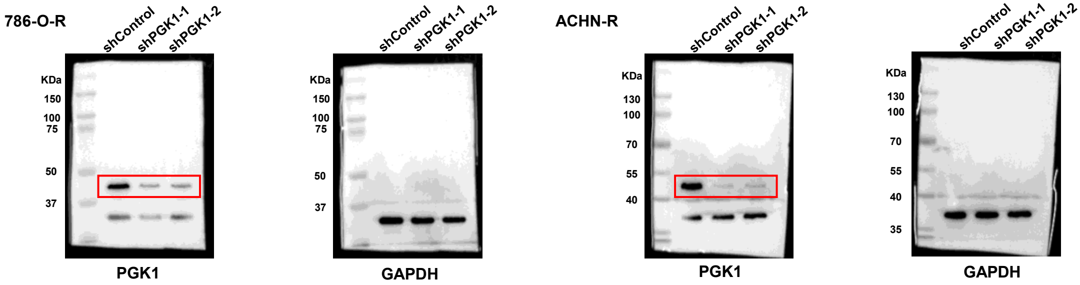

Fig. 5G

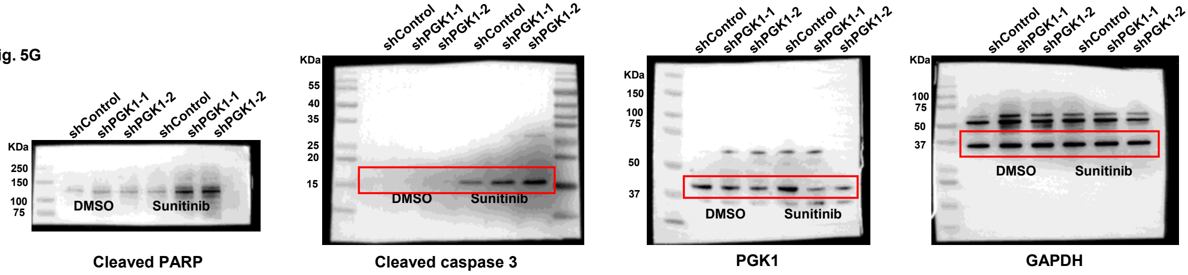

Fig. 5I

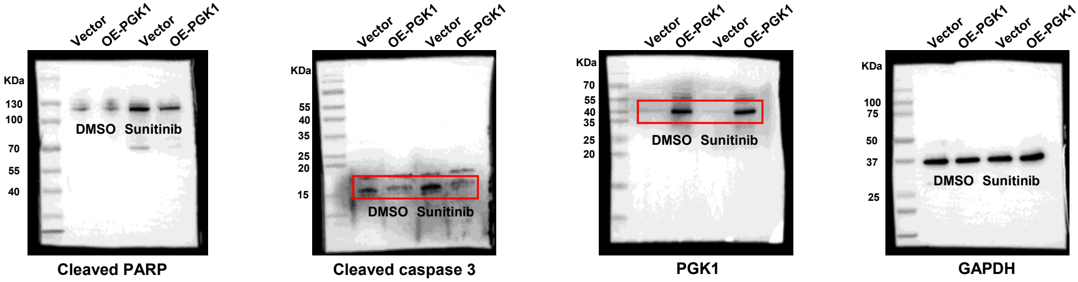

Fig. 5K

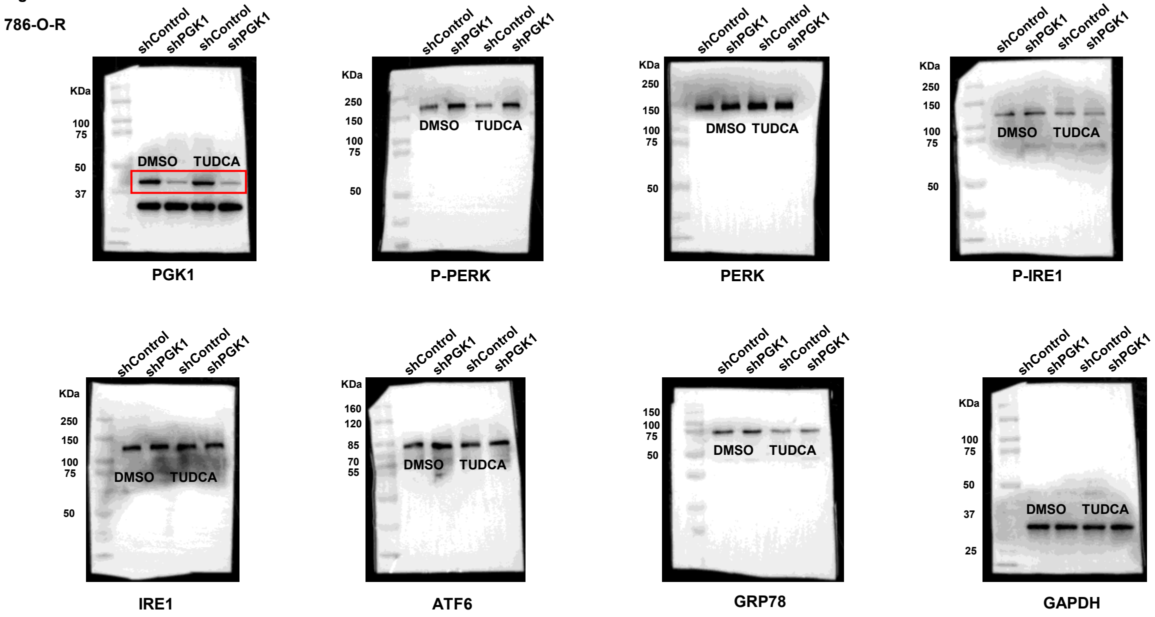

ACHN-R

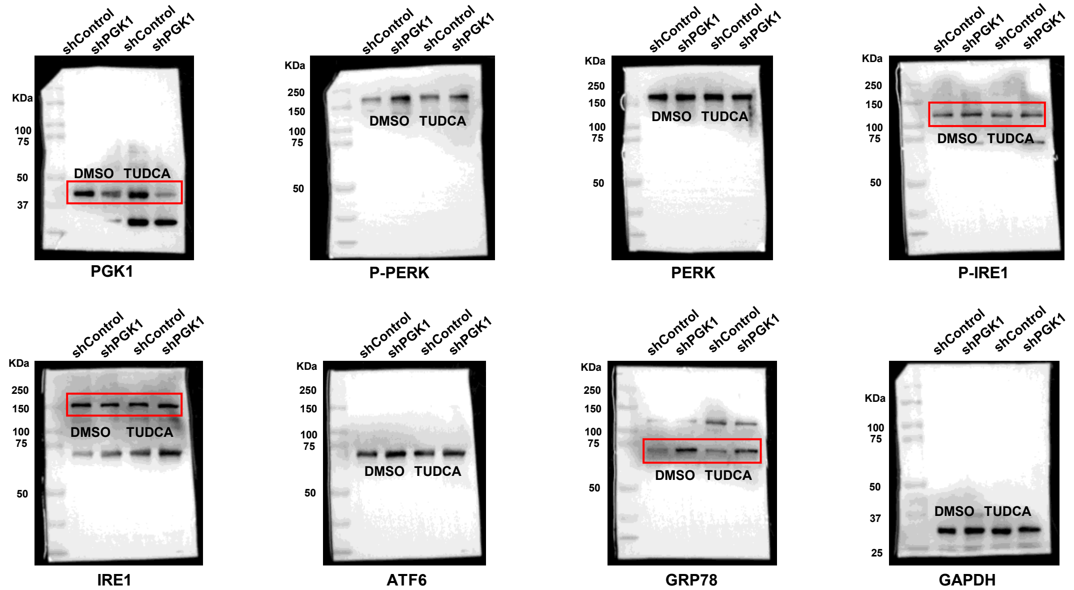

Fig. 5L

ACHN

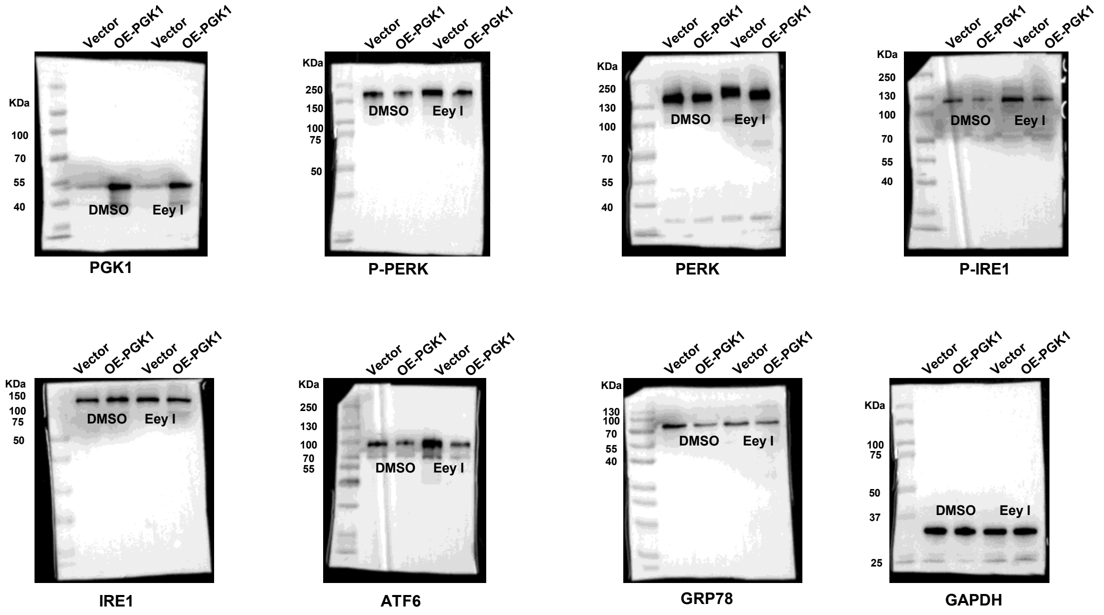

769-P

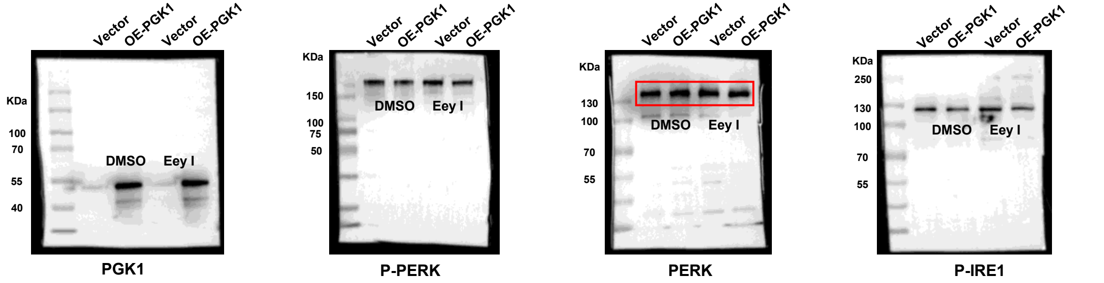

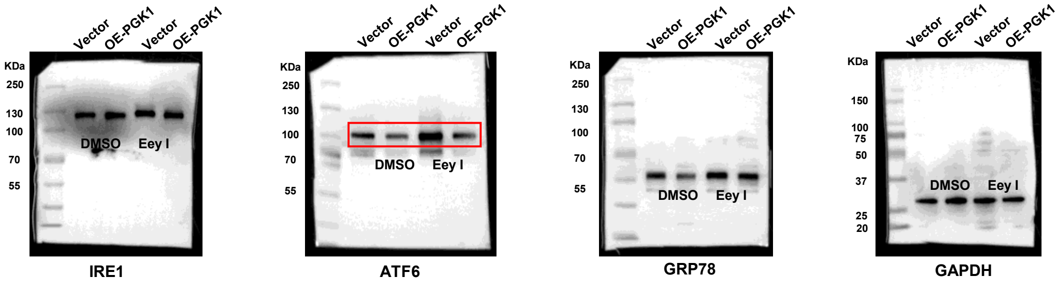

Fig. 6A

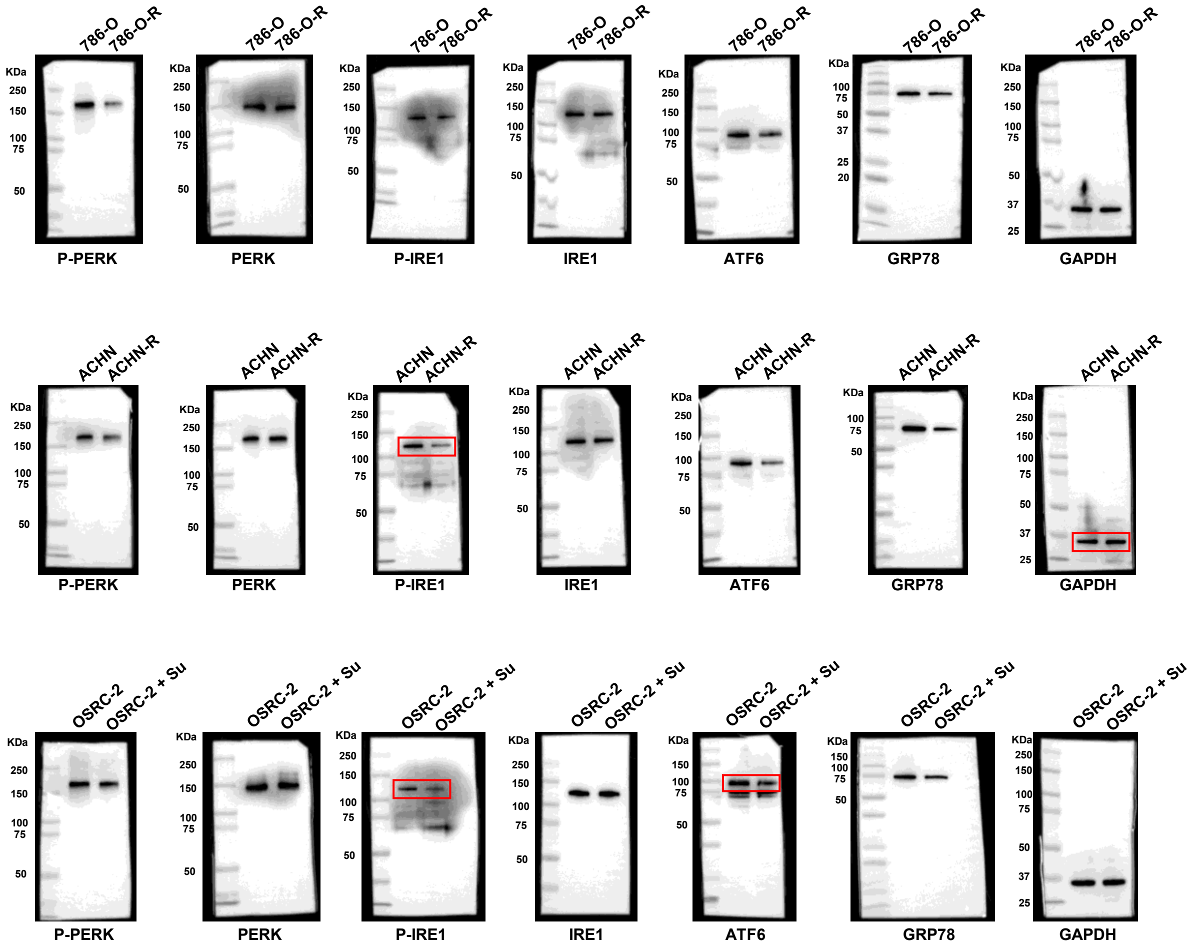

Fig. S1A

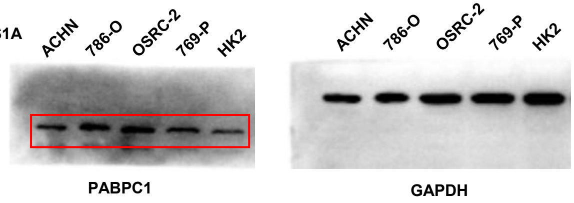

Fig. S1B

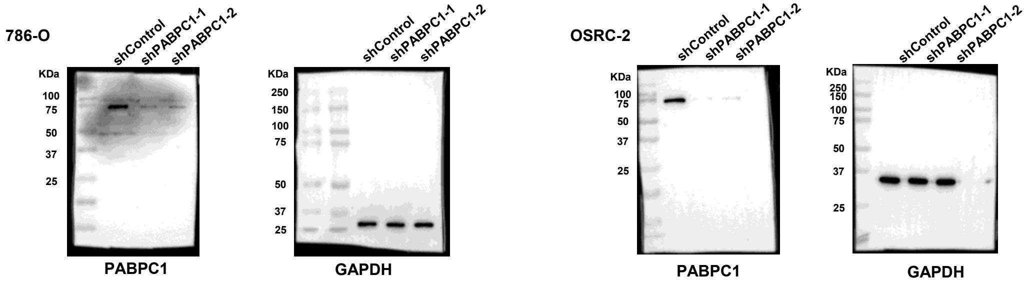

Fig. S1C

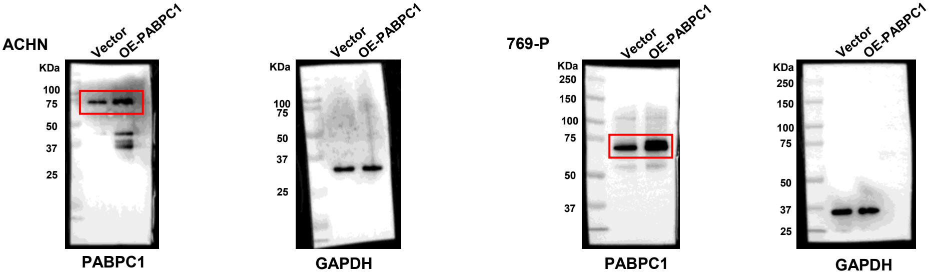

Fig. S2A

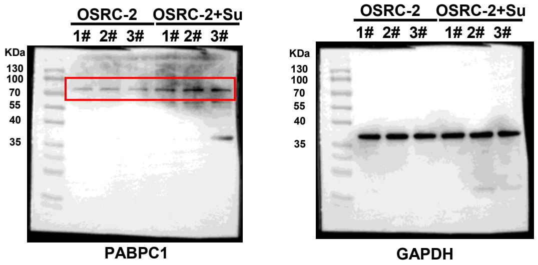

Fig. S2B

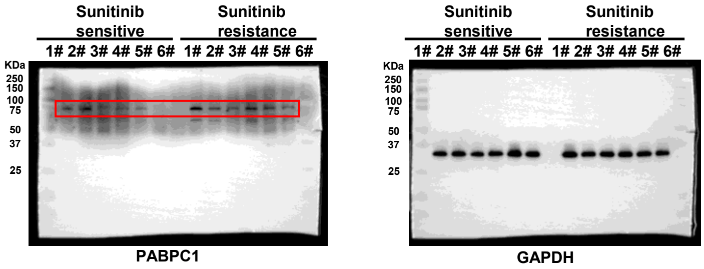

Fig. S2G

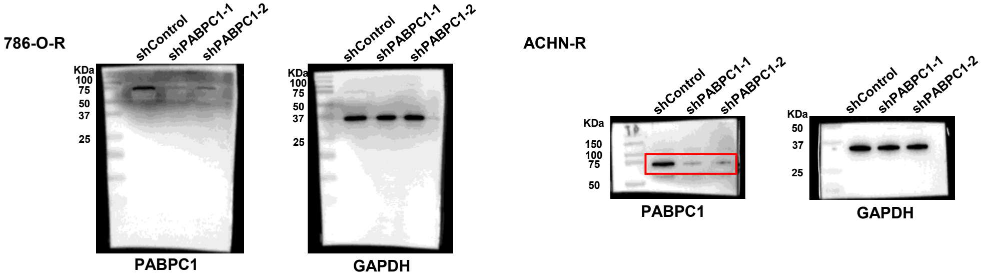

Fig. S5A

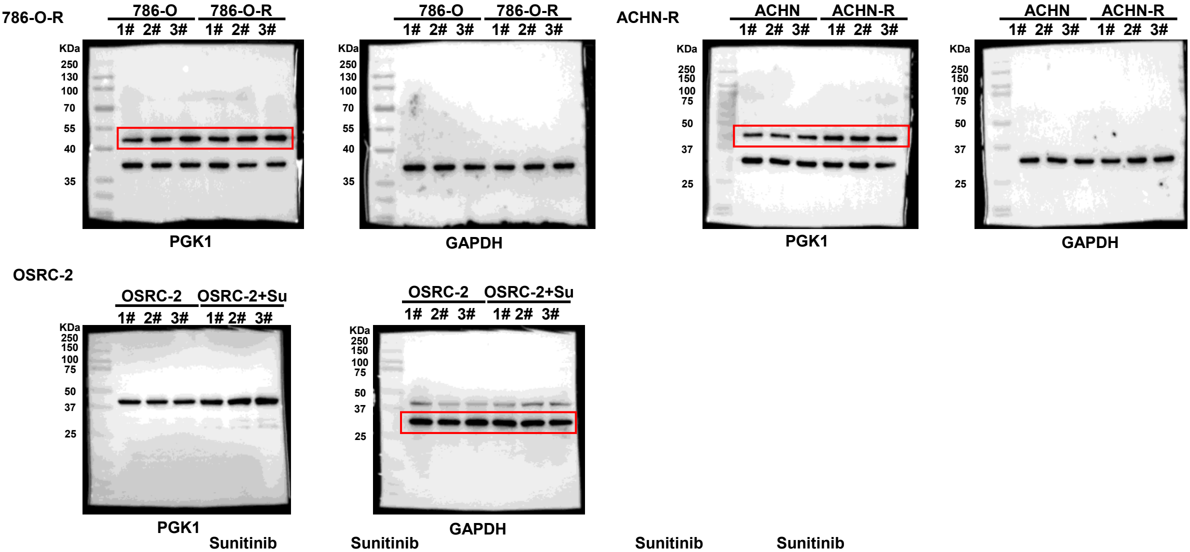

Fig. S5B

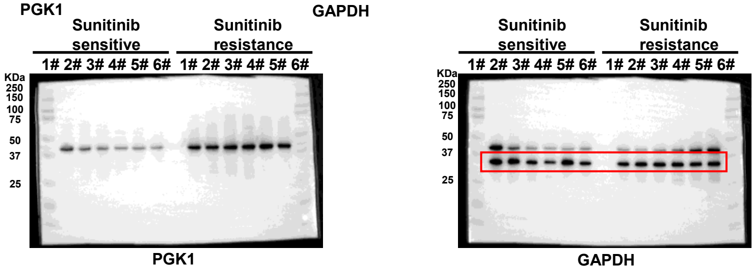

Fig. S5C

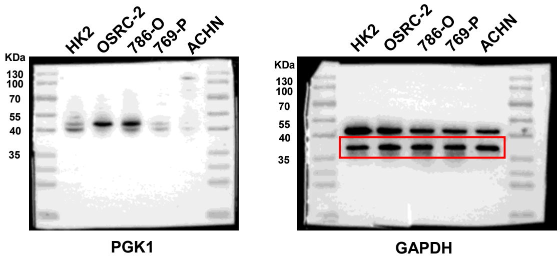

Fig. S5D

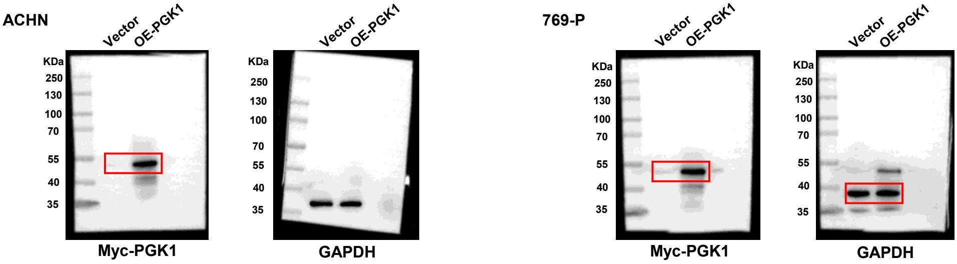

Supplement: Supplementary file 8 — Original western blots [file 41419_2026_8676_MOESM8_ESM.pdf]
